# Theophylline derivatives promote primordial follicle activation via cAMP-PI3K/Akt pathway and ameliorate fertility deficits in naturally aged mice

**DOI:** 10.7150/ijbs.99936

**Published:** 2024-09-30

**Authors:** Wenbo Zhang, Longwei Gao, Xiaodan Zhang, Yashuang Weng, Yan Du, Yan-Li Sun, Hongwei Wei, Tiantian Hao, Yuezhou Chen, Xiaoyan Liang, Meijia Zhang

**Affiliations:** 1 The Innovation Centre of Ministry of Education for Development and Diseases, The Second Affiliated Hospital, School of Medicine, South China University of Technology, Guangzhou, Guangdong, 510006, China.; 2 Reproductive Medicine Research Center, The Sixth Affiliated Hospital of Sun Yat-sen University, Guangzhou, Guangdong, 510655, China.; 3 Reproductive Medicine Center, Zhongshan City People's Hospital, Zhongshan, Guangdong, 528403, China.

**Keywords:** premature ovarian insufficiency, primordial follicle activation, phosphodiesterase, theophylline derivative, fertility

## Abstract

In elderly women and patients with premature ovarian insufficiency (POI), activating their remaining dormant primordial follicles *in vivo* is challenging. In this study, we found that phosphodiesterase (PDE) subtypes were expressed mainly in primordial follicle oocytes. The specific PDE inhibitors and theophylline derivatives (aminophylline, dyphylline, and enprofylline) activated primordial follicles in neonatal mice by ovary culture and intraperitoneal injection. These inhibitors also increased the levels of ovarian cyclic adenosine monophosphate (cAMP) and oocyte phosphorylated protein kinase B (p-Akt). The blockade of gap junctions using carbenoxolone (CBX) increased the levels of ovarian cAMP and pre-granulosa cell phosphorylated mammalian target of rapamycin (p-mTOR), suggesting that oocyte PDEs hydrolyze cAMP from pre-granulosa cells through gap junctions to maintain primordial follicle dormancy. Importantly, oral aminophylline improved ovulated oocyte quantity and quality, and increased offspring numbers in naturally aged mice. In addition, theophylline derivatives also activated human primordial follicles and increased p-Akt levels. Thus, theophylline derivatives activate primordial follicles by accumulating cAMP levels and activating phosphatidylinositol 3-kinase (PI3K)/Akt pathway in oocytes, and oral aminophylline increased fertility in naturally aged female mice by improving ovulated oocyte quantity and quality. As oral medications, theophylline derivatives may be used to improve fertility in elderly women and patients with POI.

## Introduction

The primordial follicle pool in mammals forms in the ovaries either before or shortly after birth 1. During each cycle, only a few follicles begin growth, while most remain dormant, preserving reproductive potential 2, 3. The primordial follicle is composed of several flat pre-granulosa cells surrounding a dormant oocyte 4. When primordial follicles are activated, oocytes start to grow and flat pre-granulosa cells differentiate into the cubic form 5. In pre-granulosa cells, the proto-oncogenic receptor tyrosine kinase ligand (KITL) is induced by glycolysis-dependent mammalian target of rapamycin (mTOR) signaling 6. In oocytes, KITL activates phosphatidylinositol 3-kinase (PI3K)-protein kinase B (Akt) signaling, causing forkhead box O3a (FOXO3a) to move out of the nucleus, subsequently activating primordial follicles 2, 7.

The strict regulation of primordial follicle activation is crucial for preserving female reproductive lifespan 4. The premature depletion of these follicles can result in premature ovarian insufficiency (POI) 8. Patients with POI often possess dormant primordial follicles that are difficult to activate *in vivo*, and cannot restore fertility by traditional assisted reproductive technology (ART) 9. *In vitro* activation (IVA) by using an mTOR activator and PI3K stimulators or by disrupting the Hippo pathway has been applied to treat POI patients, through which only a few patients have successfully acquired their children 10, 11. Additionally, IVA requires surgery, and stimulators increase the risk of DNA damage and cancer 12-14. All these factors limit the widespread application of IVA. Our recent research indicates that the oral administration of the metallic compound can activate residual primordial follicles in aged mice and alleviate their fertility defects 15. However, the metallic compound has no specific target, which may cause side effects during the clinical application in POI patients. Therefore, safer and more effective oral drugs are needed to alleviate the fertility defects in POI patients.

Glycolysis and PI3K/Akt activity can be promoted by cyclic adenosine monophosphate (cAMP) signaling in various tissues and cells 16-19. Cyclic AMP is produced by adenylate cyclases (ADCYs) and is hydrolyzed by phosphodiesterases (PDEs) to maintain intracellular cAMP homeostasis 20, 21. On the other hand, cyclic guanosine monophosphate (cGMP) increases cAMP levels by competitively inhibiting cAMP hydrolysis 22-24. A recent report shows that the inhibition of PDE3A activity in oocytes by milrinone increases cAMP levels and promotes mouse primordial follicle activation 25. Nevertheless, the knockout of *Pde3a* does not influence primordial follicle activation 26, suggesting that other PDE subtypes compensate for maintaining primordial follicle dormancy.

Theophylline, the nonspecific PDE inhibitor, is a natural compound extracted from tea and cocoa plants 27. Many theophylline derivatives have been synthesized to enhance the solubility and pharmaceutical activity of this compound 28. These theophylline derivatives can be divided into four categories: combinations of theophylline with different salts or bases, compounds with N7 substitution of theophylline, compounds with N3-methyl substitution of theophylline, and substitutions at other positions on theophylline. These theophylline derivatives are commonly used for treating chronic obstructive pulmonary disease (COPD) and bronchial asthma 29, 30. Thus, we hypothesized that these theophylline derivatives can promote primordial follicle activation via increasing cAMP levels.

In the present study, PDE subtypes were found to be expressed mainly in primordial follicle oocytes. Theophylline derivatives, known as PDE inhibitors, enhanced the activation of mouse and human primordial follicles through the accumulation of cAMP and the activation of PI3K/Akt pathway within oocytes. Furthermore, oral aminophylline not only increased ovulated oocyte quantity and quality but also ameliorated infertility in naturally aged female mice. Therefore, it is recommended that oral theophylline derivatives could potentially activate the remaining dormant primordial follicles in patients with POI and elderly women.

## Results

### The specific PDE inhibitors promote mouse primordial follicle activation via the oocyte PI3K/Akt pathway

**Figure 1:**
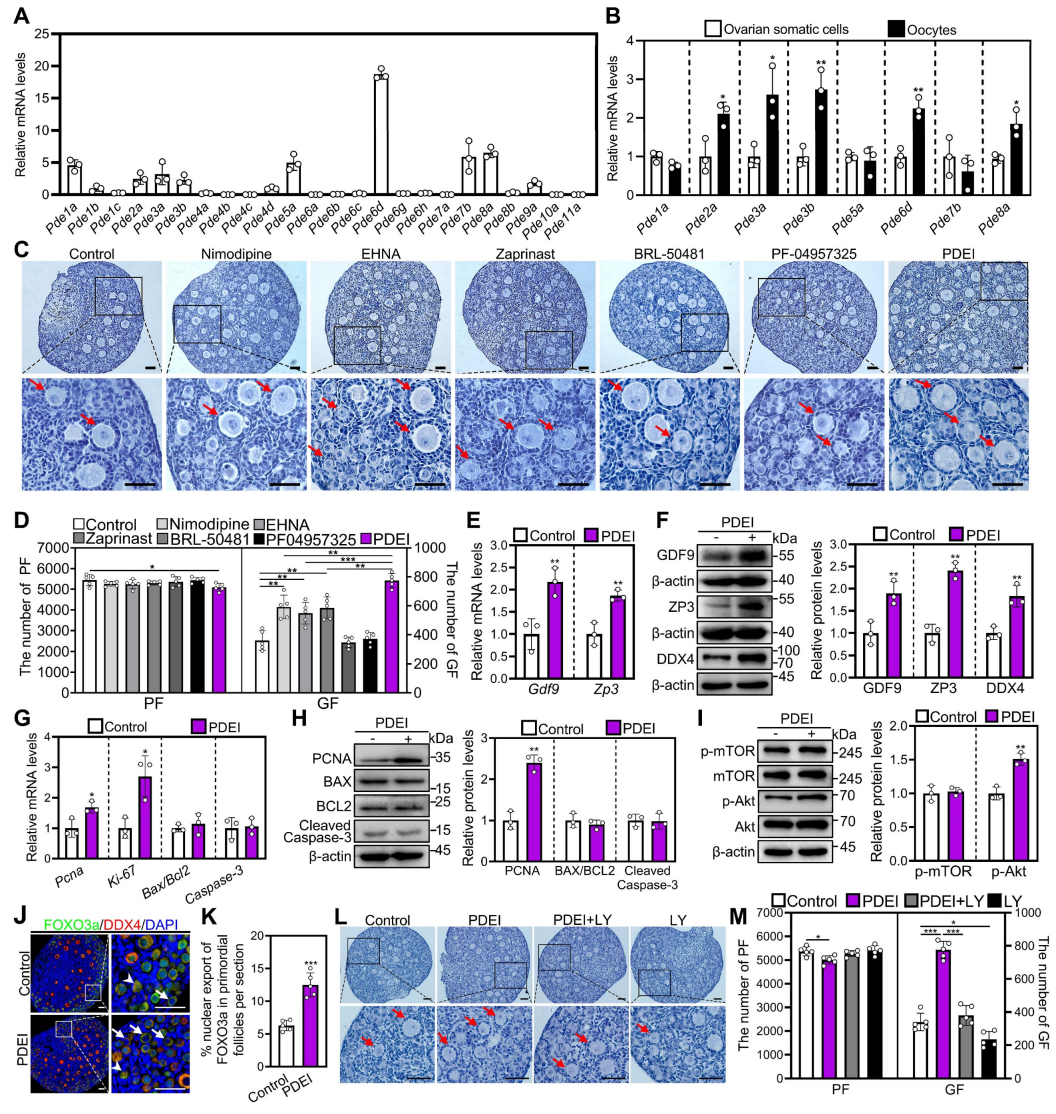
** Effects of specific PDE inhibitors on mouse primordial follicle activation *in vitro*.** 4 dpp mouse ovaries were collected for qRT‒PCR analysis (**A-B**). 3 dpp mouse ovaries were cultured in medium alone or with 10 μM nimodipine, 2.5 μM EHNA, 5 μM zaprinast, 50 μM BRL-50481, 100 nM PF-04957325, PDEI (the combination of 10 μM nimodipine, 2.5 μM EHNA, and 5 μM zaprinast), and/or 10 μM LY294002 (LY) for 1 (**G**, **I**), 2 (**E-F, H,** and **J**-**K)**, or 4 days (**C-D** and **L-M**). **A**, The phosphodiesterase subtype mRNA levels in the ovaries of mice at 4 dpp. The mRNA value of *Pde4d* was set as 1 (n = 3 biological replicates). **B**, The comparison of highly expressed phosphodiesterase subtype mRNA levels in ovarian somatic cells and oocytes of 4 dpp mouse primordial follicles. *P < 0.05 and **P < 0.01 vs. ovarian somatic cells group. **C-D**, The comparison of ovarian morphology (**C**) and primordial and growing follicle number (**D**) across various groups. The ovarian sections were hematoxylin-stained. **E**, The comparison of *Gdf9* and *Zp3* mRNA levels between control and PDEI groups. **F**, The comparison of GDF9, ZP3, and DDX4 protein (**F**) levels between control and PDEI groups. **G-H**, The comparison of PCNA, BAX, BCL-2, Cleaved Caspase-3, and Ki-67 mRNA (**G**) and/or protein (**H**) levels between control and PDEI groups**. I**, The comparison of p-mTOR and p-Akt protein levels between control and PDEI groups. **J-K**, FOXO3a localization in primordial follicle oocyte nuclear (arrowheads) or cytoplasm (arrows. **J**) and the comparison of FOXO3a nuclear export percentage in primordial follicle oocytes (**K**) between control and PDEI groups. DDX4, red; FOXO3a, green. **L-M**, The comparison of ovarian morphology (**L**) and primordial and growing follicle number (**M**) across various groups. The representative images were displayed. PF, primordial follicle; GF, growing follicle. Red arrows, growing follicles. PF, primordial follicle; GF, growing follicle. Scale bars, 50 µm. In each experiment, n ≥ 3 biological replicates. Bars indicate the mean ± SD. *P < 0.05, **P < 0.01, and ***P < 0.001. Two-tailed unpaired t-test was used to assess statistical significance.

First, we studied the expression of PDE subtypes in the ovaries of mice at 4 days postpartum (dpp), in which almost all follicles are primordial follicles. *Pde6d* was the most highly expressed PDE subtype, followed by *Pde1a*, *Pde2a*, *Pde3a*, *Pde3b*, *Pde5a*, *Pde7b*, and *Pde8a* (Figure 1A). *Pde1b*, *Pde4d*, and *Pde9a* were slightly expressed, and *Pde1c*, *Pde4a*, *Pde4b*, *Pde4c*, *Pde6a*, *Pde6b*, *Pde6c*, *Pde6g*, *Pde6h*, *Pde7a*, *Pde8b*, *Pde10a*, and *Pde11a* were barely expressed (Figure 1A). Next, we analyzed the localization of the highly expressed PDE subtypes in 4 dpp mouse ovaries. *Pde2a*, *Pde3a*, *Pde3b*, *Pde6d*, and *Pde8a* were mainly expressed in the oocytes of mouse primordial follicles (Figure 1B). The highly expressed PDE subtypes were further analyzed in mouse ovaries at 1, 4, and 7 dpp. *Pde1a* and *Pde2a* mRNA levels were significantly decreased, while *Pde3a*, *Pde3b*, *Pde5a*, *Pde6d*, *Pde7b*, and *Pde8a* mRNA levels did not show significant changes during primordial follicle activation (Figure S1A).

Then, we used specific inhibitors targeting these highly expressed PDE subtypes to investigate their impact on the activation of primordial follicles 31-34. The ovaries of neonatal mice were treated with these inhibitors for 4 days. Compared with control, the PDE1 inhibitor nimodipine, PDE2 inhibitor EHNA, and PDE5/6 inhibitor zaprinast dose-dependently increased growing follicle numbers, with optimal concentrations of 10, 2.5, and 5 μM, respectively (Figure S1B-D). However, BRL-50481 (a PDE7 inhibitor, 0-50 μM) and PF04957325 (a PDE8 inhibitor, 0-10 nM) showed no impact on growing follicle numbers (Figure S1E and S1F). PDE6D is involved in primordial follicle activation, although *Pde6d* does not show significant changes during primordial follicle activation. Compared to a single PDE inhibitor nimodipine, EHNA, or zaprinast, their combination (named PDEI) further increased growing follicle numbers (Figure 1C and 1D), suggesting that these PDE subtypes compensate for each other in regulating primordial follicle activation. Furthermore, PDEI significantly increased growth differentiation factor 9 (GDF9), zona pellucida glycoprotein 3 (ZP3), and DEAD-box helicase 4 (DDX4) mRNA and/or protein levels compared with control (Figure 1E and 1F). Moreover, PDEI significantly increased Ki-67 and proliferating cell nuclear antigen (PCNA) mRNA and/or protein levels, the proportion of signals for Ki-67, PCNA, and/or bromodeoxyuridine (BrdU) in primordial follicles and granulosa cells, as well as the count of BrdU signals in somatic cells (Figure 1G and 1H, Figure S2A-D). Nevertheless, PDEI showed no impact on B-cell lymphoma 2-associated X (BAX)/B-cell lymphoma 2 (BCL-2) or Cleaved Caspase-3 levels, or Cleaved Caspase-3-positive cell numbers compared with the control (Figure 1G and 1H, Figure S2C). Thus, these specific PDE inhibitors promote the activation of mouse primordial follicles *in vitro*.

The impact of PDEI on the p-mTOR and p-Akt levels was investigated using western blotting and immunofluorescence analysis. Compared with control, PDEI significantly increased p-Akt levels in ovaries and its fluorescence intensity in oocytes (Figure 1I, Figure S3A-C). Consistent with these findings, PDEI also significantly increased the percentage of primordial follicle oocytes exporting FOXO3a from the nucleus compared to the control group (Figure 1J and 1K). Treatment with LY294002, a PI3K/Akt inhibitor, blocked the PDEI-induced primordial follicle activation (Figure 1L and 1M). Therefore, PDEI promotes mouse primordial follicle activation via oocyte PI3K/Akt pathway.

### PDEI increases the accumulation of cAMP by inhibiting oocyte PDE activity

We assessed the impact of PDEI on cyclic nucleotide levels. PDEI significantly increased cAMP levels but not cGMP levels in cultured mouse ovaries compared with control (Figure S4E and S4F). Consistent with a previous study 25, dibutyryl cAMP (dbcAMP) significantly increased growing follicle number compared with control (Figure S4A and S4B). Thus, PDEI activates primordial follicles by elevating ovary cAMP levels. 8-bromo-cGMP (8br-cGMP) also significantly increased growing follicle number (Figure S4A and S4C) possibly by inhibiting the hydrolysis of cAMP 22-24.

**Figure 2:**
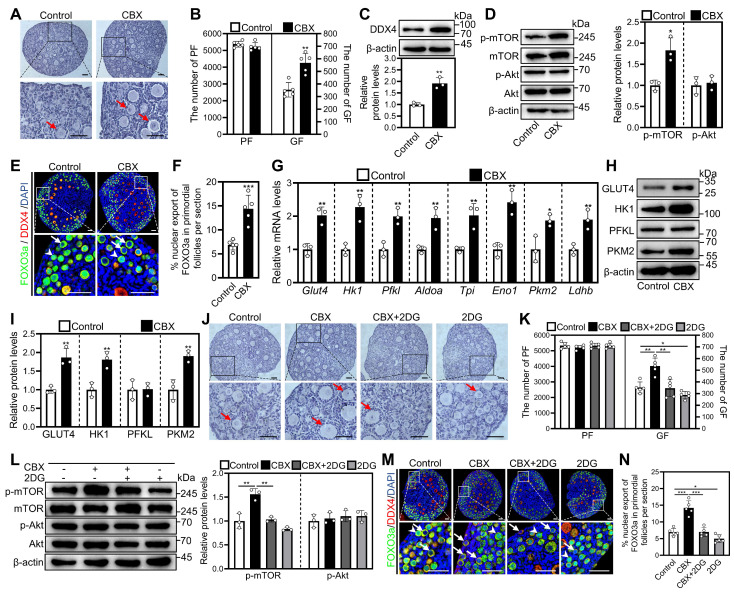
** Effects of CBX on mouse primordial follicle activation *in vitro*.** 3 dpp mouse ovaries were cultured in medium alone or with 10 μM CBX and/or 5 mM 2DG for 1 (**D, G, and L**), 2 (**C**, **E-F, H-K, and M-N**), or 4 days (**A-B**). **A-B**, The comparison of ovarian morphology (**A**) and primordial and growing follicle number (**B**) between control and CBX groups. **C-D**, The comparison of DDX4 (**C**), p-mTOR and p-Akt (**D**) protein levels between control and CBX groups. **E-F**, FOXO3a localization in primordial follicle oocyte nuclear (arrowheads) or cytoplasm (arrows. **E**) and the comparison of FOXO3a nuclear export percentage in primordial follicle oocytes (**F**) across various groups. **G,** The comparison of *Glut4, Hk1, Pfkl, Aldoa, Tpi, Eno1, Pkm2*, and* Ldhb* mRNA levels between control and CBX groups. **H-I**, The comparison of HK1, PFKL, PKM2, and GLUT4 protein levels between control and CBX groups. **J-K**, The comparison of ovarian morphology (**J**) and primordial and growing follicle number (**K**) across various groups. **L**, The comparison of p-mTOR and p-Akt protein levels between control and CBX groups. **M-N**, FOXO3a localization in primordial follicle oocyte nuclear (arrowheads) or cytoplasm (arrows. **M**) and the comparison of FOXO3a nuclear export percentage in primordial follicle oocytes (**N**) across various groups. Red arrows, growing follicles. The ovarian sections were hematoxylin-stained. DDX4, red; FOXO3a green. The representative images were displayed. PF, primordial follicle; GF, growing follicle. Scale bars, 50 µm. In each experiment, n ≥ 3 biological replicates. Bars indicate the mean ± SD. *P < 0.05, **P < 0.01, and ***P < 0.001. Two-tailed unpaired t-test was used to assess statistical significance.

We next analyzed the role of gap junctions, through which granulosa cell-produced cAMP can enter oocytes to perform physiological functions 22. The blockade of gap junctions by carbenoxolone (CBX) increased growing follicle number and cAMP levels in cultured mouse ovaries (Figure 2A and 2B, Figure S4D-F). Consistent with this, CBX significantly increased DDX4 protein levels (Figure 2C) and the proportion of signals for Ki-67, PCNA, and/or BrdU in primordial follicles and granulosa cells, as well as the count of BrdU signals in somatic cells (Figure S5A-D). Nevertheless, CBX showed no impact on Cleaved Caspase-3-positive cell numbers compared with control (Figure S5A and S5C).

In addition, CBX significantly increased p-mTOR levels in ovaries and fluorescence intensity in granulosa cells compared with control (Figure 2D, Figure S3A-C). Consistent with these findings, CBX significantly increased the percentage of primordial follicle oocytes exporting FOXO3a from the nucleus compared with control (Figure 2E and 2F). Moreover, CBX significantly increased the levels of mRNA related to glycolysis (aldolase A (*Aldoa*), enolase 1 (*Eno1*), glucose transporters type 4 (*Glut4*), hexokinase 1 (*Hk1*), lactate dehydrogenase B (*Ldhb*), phosphofructokinase liver type (*Pfkl*), pyruvate kinase M2 (*Pkm2*), and triosephosphate isomerase (*Tpi*)) as well as the corresponding proteins (including GLUT4, HK1, and PKM2) compared with control (Figure 2G-I). These effects of CBX were blocked by the glycolysis inhibitor 2-DG (Figure 2J-N). These results suggest that CBX increases the accumulation of cAMP in pre-granulosa cells by preventing its entry into oocytes and subsequent degradation. Thus, PDEI increases the accumulation of cAMP by inhibiting oocyte PDE activity during mouse primordial follicle activation.

### The nonspecific PDE inhibitors theophylline derivatives promote mouse primordial follicle activation *in vitro*

**Figure 3:**
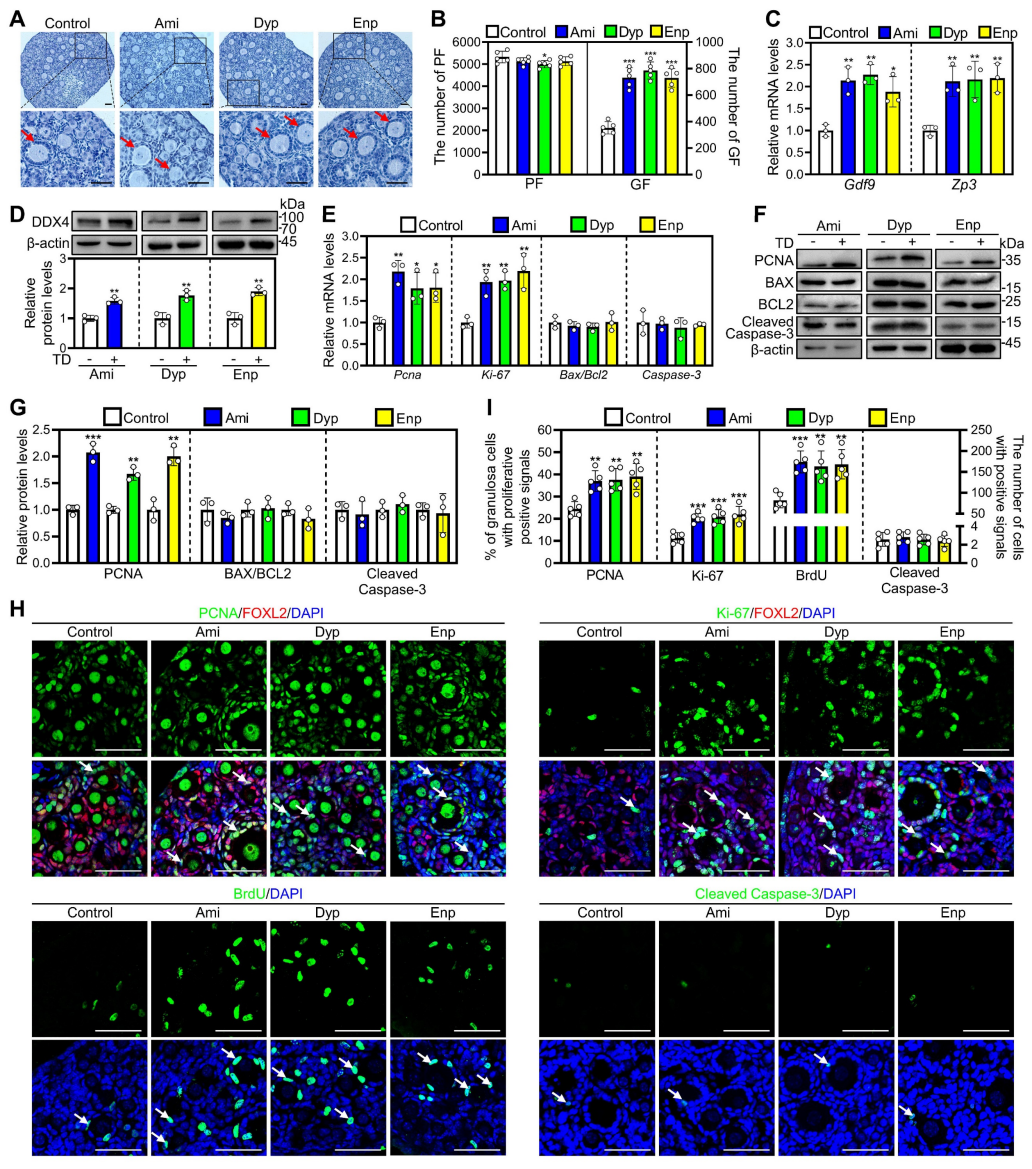
**Effects of theophylline derivatives on mouse primordial follicle activation *in vitro*.** 3 dpp mouse ovaries were cultured in medium alone or with 50 μM aminophylline (Ami), 160 μM dyphylline (Dyp), or 10 μM enprofylline (Enp) for 1 (**E**), 2 (**C-D** and **F-I**), or 4 days (**A-B**). **A-B**, The comparison of ovarian morphology (**A**) and primordial and growing follicle number (**B**) across various groups. The ovarian sections were hematoxylin-stained. **C-D**, The comparison of *Gdf9* and *Zp3* mRNA (**C**) and DDX4 protein (**D**) levels across various groups. **E-G**, The comparison of PCNA, BAX, BCL-2, Cleaved Caspase-3, and Ki-67 mRNA (**E**) and/or protein (**F**, **G**) levels across various groups. **H**, PCNA, Ki-67, BrdU, and Cleaved Caspase-3 immunofluorescence stain (green) across various groups. FOXL2, red. **I**, The comparison of PCNA- and Ki-67-positive granulosa cell percentage and BrdU- and Cleaved Caspase-3-positive cell number across various groups. The representative images were displayed. Red arrows, growing follicles. PF, primordial follicle; GF, growing follicle. TD, theophylline derivatives. Scale bars, 50 µm. In each experiment, n ≥ 3 biological replicates. Bars indicate the mean ± SD. *P < 0.05, **P < 0.01, and ***P < 0.001. Two-tailed unpaired t-test was used to assess statistical significance.

Since many PDE subtypes are expressed in primordial follicular oocytes, we used nonspecific PDE inhibitors for further investigation. As nonspecific PDE inhibitors, the representative theophylline derivatives from each category were selected to study their impact on mouse primordial follicles activation. The results showed that aminophylline, dyphylline, and enprofylline dose-dependently increased growing follicle numbers, and the optimal concentrations were 50, 160, and 10 μM, respectively (Figure S6A-D). In addition, aminophylline time-dependently promoted mouse primordial follicle activation (Figure S6E-G), suggesting an accumulated effect. Furthermore, treatment with aminophylline, dyphylline, and enprofylline significantly increased cAMP levels, growing follicle numbers, *Gdf9* and *Zp3* mRNA and DDX4 protein levels compared with control (Figure 3A-D, Figure S4E and S4F). All three of these theophylline derivatives also significantly increased Ki-67 and PCNA mRNA and/or protein levels, the proportion of signals for Ki-67 and PCNA in granulosa cells, and the count of BrdU signals in somatic cells (Figure 3E-I, Figure S7). Nevertheless, these theophylline derivatives showed no impact on BAX/BCL-2 or Cleaved Caspase-3 levels, or Cleaved Caspase-3-positive cell numbers compared with control (Figure 3E-I, Figure S7). Thus, these theophylline derivatives promote mouse primordial follicle activation via increasing cAMP levels. cAMP acts through protein kinase A (PKA), exchange protein activated by cAMP (EPAC), and hyperpolarization-activated cyclic nucleotide-gated (HCN) channels pathways 35. H89 (a PKA inhibitor), ESI09 (an EPAC inhibitor), and ivabradine (a HCN inhibitor) blocked aminophylline-promoted mouse primordial follicle activation (Figure S8A-C), suggesting that the involvement of these three pathways in cAMP-mediated primordial follicle activation.

### Theophylline derivatives promote mouse primordial follicle activation via the PI3K/Akt pathway in oocytes

**Figure 4:**
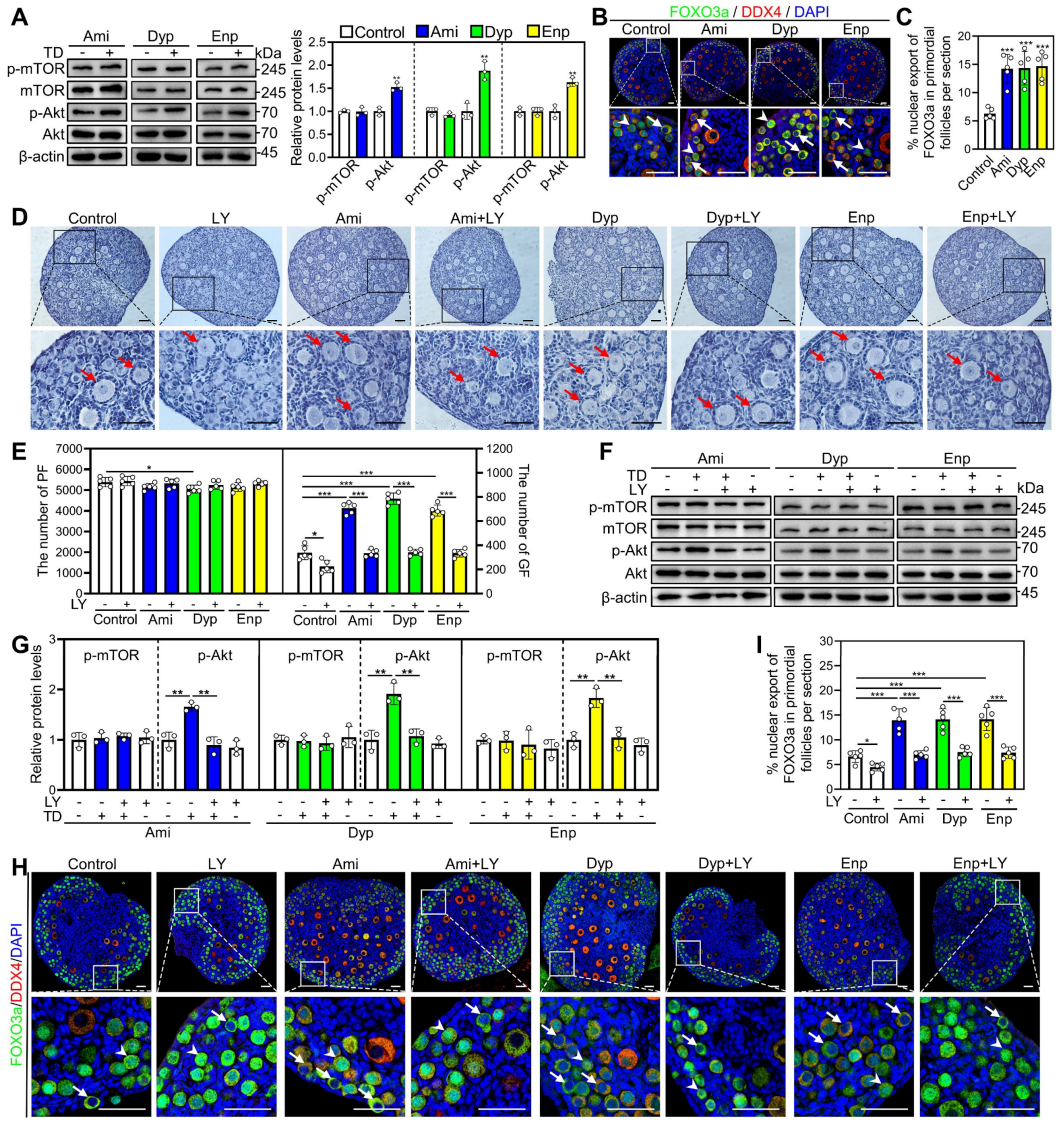
** Effects of theophylline derivatives on the PI3K/Akt pathway activation *in vitro*.** 3 dpp mouse ovaries were cultured in medium alone or with 50 μM aminophylline (Ami), 160 μM dyphylline (Dyp), 10 μM enprofylline (Enp), and/or 10 μM LY294002 (LY) for 1 (**A** and **F-G**), 2 (**B-C** and **H-I)**, or 4 days (**D-E). A**, The comparison of p-mTOR and p-Akt protein levels across various groups. **B-C**, FOXO3a localization in primordial follicle oocyte nuclear (arrowheads) or cytoplasm (arrows. **B**) and the comparison of FOXO3a nuclear export percentage in primordial follicle oocytes (**C**) across various groups. **D-E**, The comparison of ovarian morphology (**D**) and primordial and growing follicle number (**E**) across various groups. The ovarian sections were hematoxylin-stained. **F-G**, The comparison of p-mTOR and p-Akt protein levels across various groups. **H-I**, FOXO3a localization in primordial follicle oocyte nuclear (arrowheads) or cytoplasm (arrows. **H**) and the comparison of FOXO3a nuclear export percentage in primordial follicle oocytes (**I**) across various groups. DDX4, red; FOXO3a, green. The representative images were displayed. PF, primordial follicle; GF, growing follicle. Red arrows, growing follicles. Scale bars, 50 µm. In each experiment, n ≥ 3 biological replicates. Bars indicate the mean ± SD. *P < 0.05, **P < 0.01, and ***P < 0.001. Two-tailed unpaired t-test was used to assess statistical significance.

The impact of aminophylline, dyphylline, and enprofylline on p-mTOR and p-Akt levels was investigated through western blotting and immunofluorescence analysis. Compared with control, all three of these theophylline derivatives significantly increased p-Akt levels in ovaries and fluorescence intensity in oocytes (Figure 4A, Figure S3A-C). Consistent with these findings, the percentage of primordial follicle oocytes exporting FOXO3a from the nucleus were significantly increased in aminophylline, dyphylline, and enprofylline groups compared with control (Figure 4B and 4C). These effects of the theophylline derivatives were blocked by LY294002 (Figure 4D-I). Aminophylline-induced primordial follicle activation was also blocked by another PI3K/Akt inhibitor PI3K-IN-1 (Figure S8D and S8E). The results indicate that theophylline derivatives promote primordial follicle activation through the PI3K/Akt pathway in oocytes.

### Theophylline derivatives promote mouse primordial follicle activation *in vivo*

**Figure 5:**
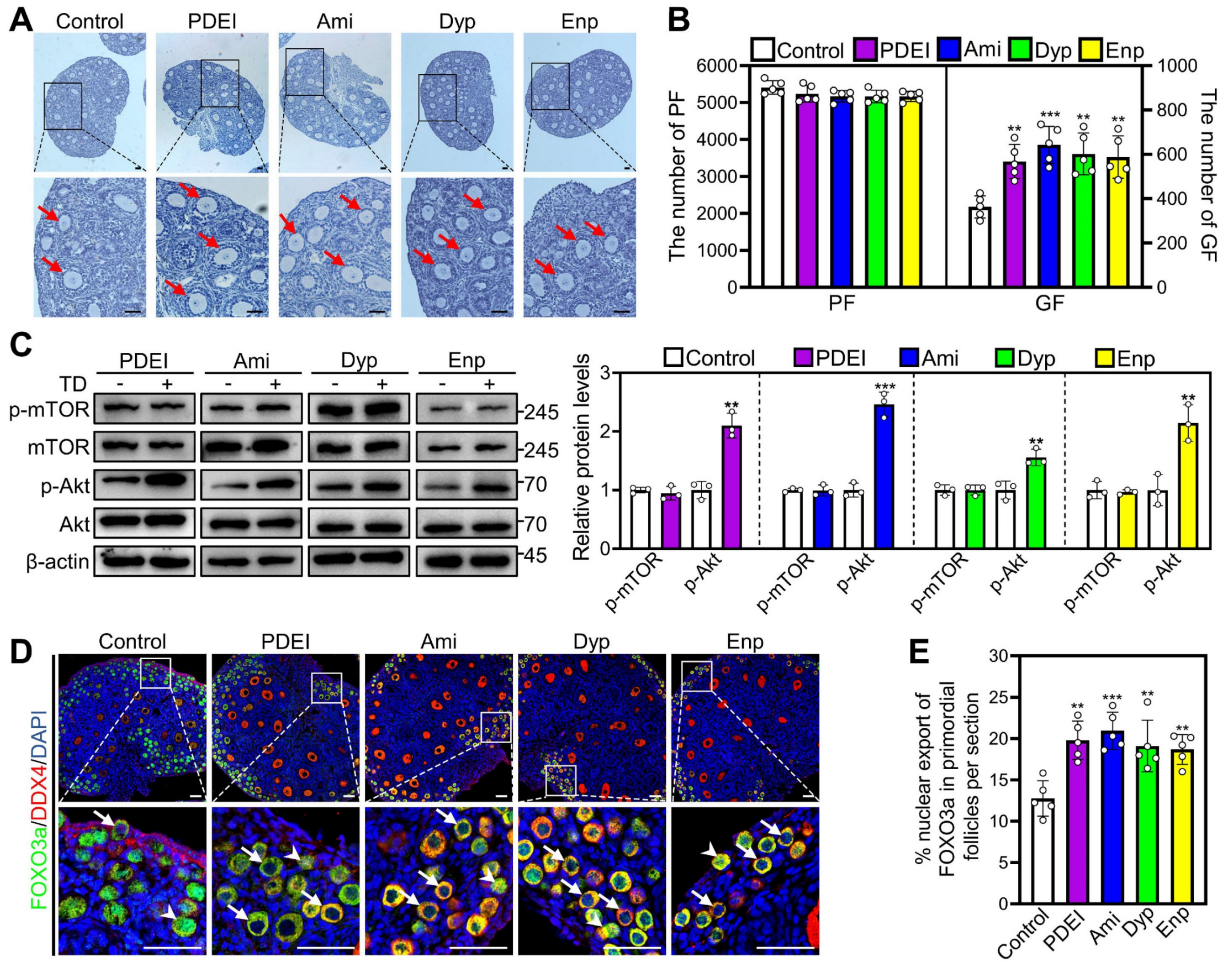
** Effects of PDEI and theophylline derivatives on mouse primordial follicle activation *in vivo*.** 3 dpp female mice were intraperitoneally injected twice a day with physiological saline, PDEI (the combination of 4.18 mg/kg nimodipine, 0.79 mg/kg EHNA, and 1.36 mg/kg zaprinast), aminophylline (Ami, 21.02 mg/kg), dyphylline (Dyp, 40.68 mg/kg), or enprofylline (Enp, 1.94 mg/kg) for two consecutive days. The ovaries were collected after 12 h (**C-E**) or 2 days (**A-B**). **A-B**, The comparison of ovarian morphology (**A**) and primordial and growing follicle (GF) number (**B**) across various groups. The ovarian sections were hematoxylin-stained. **C**, The comparison of p-mTOR and p-Akt protein levels across various groups. **D-E**, FOXO3a localization in primordial follicle oocyte nuclear (arrowheads) or cytoplasm (arrows. **D**) and the comparison of FOXO3a nuclear export percentage in primordial follicle oocytes (**E**) across various groups. DDX4, red; FOXO3a, green. The representative images were displayed. PF, primordial follicle; GF, growing follicle. Red arrows, growing follicles. Scale bars, 50 µm. In each experiment, n ≥ 3 biological replicates. Bars indicate the mean ± SD. **P < 0.01 and ***P < 0.001. Two-tailed unpaired t-test was used to assess statistical significance.

We also studied the impacts of PDE inhibitors on mouse primordial follicle activation *in vivo*. Compared with control, two daily intraperitoneal injections of PDEI, aminophylline, dyphylline, and enprofylline for two consecutive days in 3 dpp female mice significantly increased growing follicle numbers, p-Akt levels, and the percentage of primordial follicle oocytes exporting FOXO3a from the nucleus (Figure 5A-E). Furthermore, oral administration with different concentrations of theophylline derivatives promoted adolescent mouse primordial follicle activation, and the most effective concentrations were 4.0, 13.0, and 0.8 mM for aminophylline, dyphylline, and enprofylline, respectively (Figure S9A and S9B). All of these theophylline derivatives significantly increased growing follicle numbers but showed no impact on the total follicle numbers compared with control (Figure S9A and S9B). The oral administration of theophylline derivatives also had no obvious effects on ovaries, spleen, liver, small intestine, or kidney morphologies, or mouse body weight (Figure S9C-E). These findings suggest that theophylline derivatives can promote mouse primordial follicle activation *in vivo* through PI3K/Akt pathway.

### Oral aminophylline ameliorates infertility in naturally aged female mice

**Figure 6:**
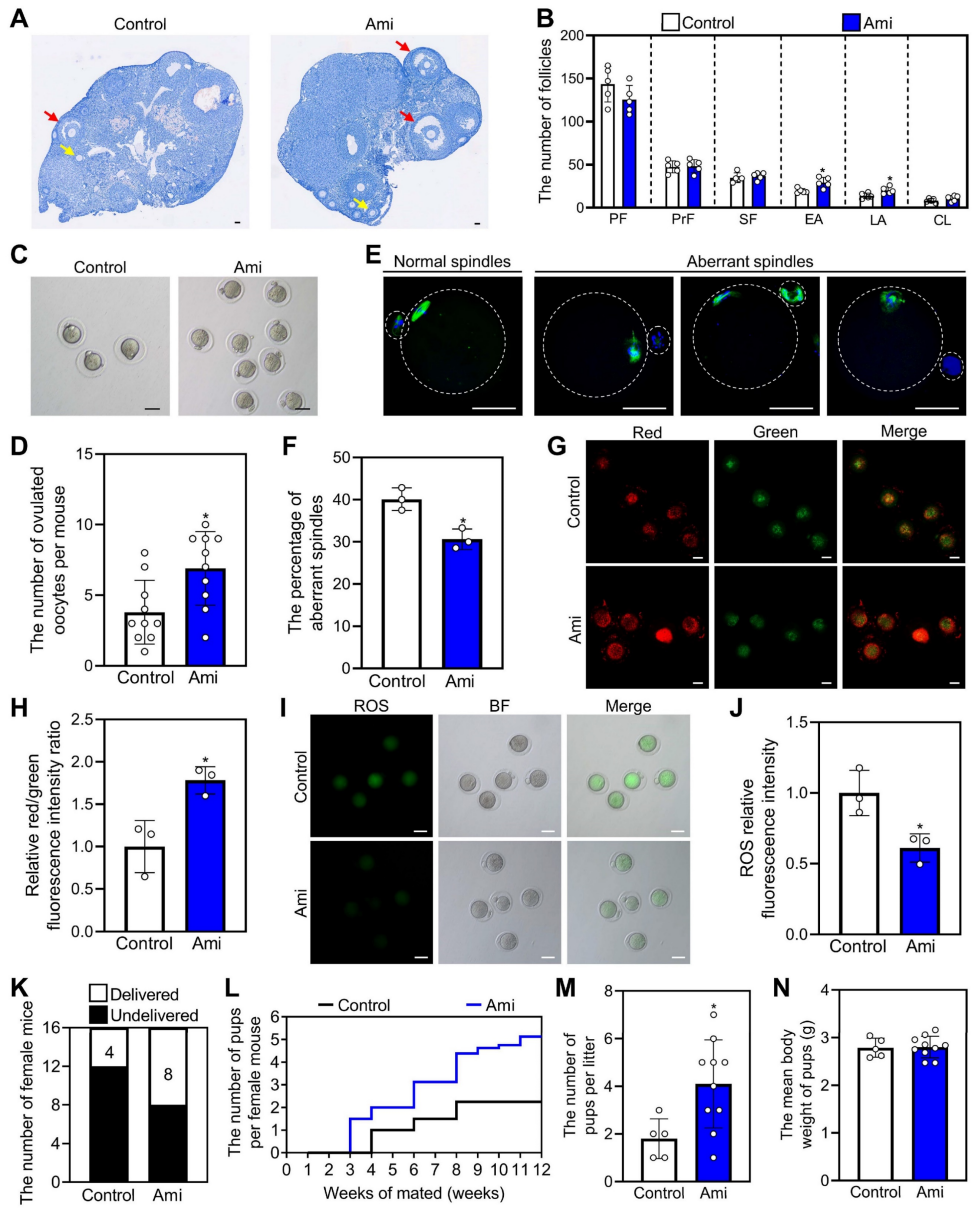
** Effects of oral administration of aminophylline on the fertility of naturally aged female mice.** The naturally aged mice were administered water either with or without 4.0 mM aminophylline, and then the fertility was examined. **A-B**, The comparison of ovarian morphology (**A**) and follicle number at different stages (**B**) between control and aminophylline groups. The ovarian sections were hematoxylin-stained. Yellow arrows, early antral follicles; red arrows, late antral follicles. **C-D**, The comparison of ovulated oocytes between control and aminophylline groups. **E**, Normal and aberrant spindles morphology. **F**, The comparison of aberrant spindles proportion between control and aminophylline groups. **G-H**, JC-1 staining showed the ΔΨm of oocytes (**G**) and the comparison of relative red/green fluorescence intensity ratio (**H**) between control and aminophylline groups. Red, higher ΔΨm; green, lower ΔΨm. **I-J**, The ROS (green) levels of oocytes (**I**) and the comparison of ROS relative fluorescence intensity (**J**) between control and aminophylline groups. Green, ROS; BF, bright field. **K-M**, The comparison of different fertility status (**K**), pups per female (**L**), and pups per litter (**M**) number in naturally aged mice between control and aminophylline groups. **N**, The comparison of pups per litter mean body weight between control and aminophylline groups. The representative images were displayed. PF, primordial follicle; PrF, primary follicle; SF, secondary follicle; EA, early antral follicle; LA, late antral follicle; CL, corpus luteum. Scale bars, 50 µm. In each experiment, n ≥ 3 biological replicates. Bars indicate the mean ± SD. *P < 0.05. Two-tailed unpaired t-test was used to assess statistical significance.

We used 14-month-old mice as a naturally aged mouse model, which is very similar to the low-fertility state seen in POI patients. The naturally aged mice were administered water either with or without 4.0 mM aminophylline for one week, followed by a three-week period of water-only intake. Compared with control, aminophylline significantly increased early and late antral follicle numbers (Figure 6A and 6B). Aminophylline also significantly increased ovulated oocyte numbers and oocyte mitochondrial membrane potential (ΔΨm), and significantly decreased aberrant spindle proportion, and reactive oxygen species (ROS) levels in oocytes (Figure 6C-J). Each group consisted of 16 female mice for three-month mating trials, and 4 mice in control and 8 mice in aminophylline group delivered 9 and 41 pups, respectively (Figure 6K and 6L). The mean number of pups per mouse and the litter count were also significantly increased in the aminophylline group compared with the control group (Figure 6L and 6M). The newborn mice in the aminophylline group had no obvious malformations, and their weights were similar to those of the control (Figure 6N). Thus, oral aminophylline increases oocyte quantity and quality by promoting primordial follicle activation and enhancing oocyte mitochondrial function.

### Oral aminophylline increases the levels of phospholipid metabolites in granulosa cells and alters regulatory pathways in oocytes

**Figure 7:**
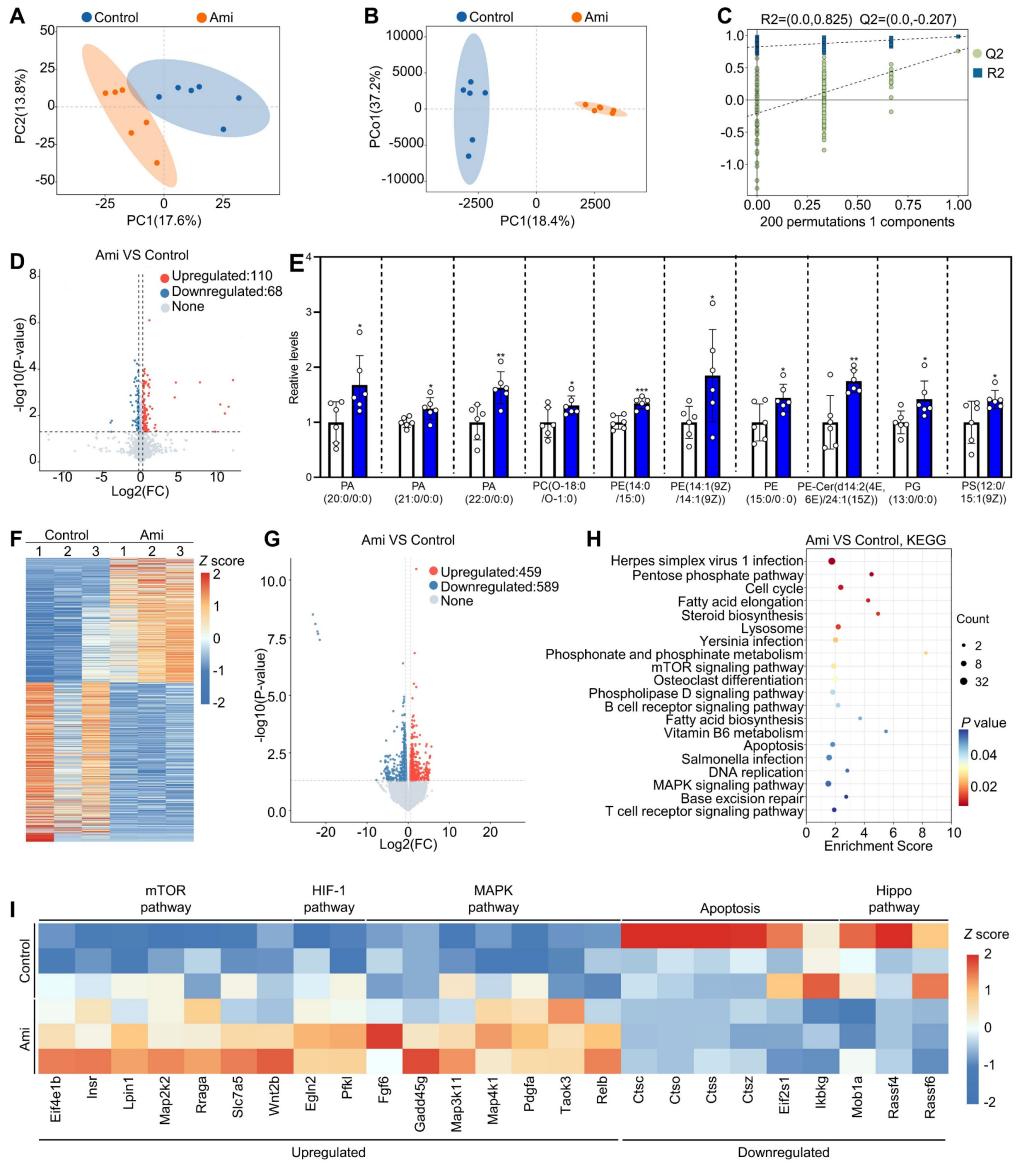
** Effects of aminophylline on the metabolome and transcriptome of naturally aged mice.** The naturally aged mice were administered water either with or without 4.0 mM aminophylline, and then the metabolome of granulosa cells (**A-E**) and transcriptome of oocytes (**F-I**) were examined. **A-B**, Principal-component analysis (PCA, **A**) and Orthogonal partial least squares-discriminant analysis (OPLS-DA, **B**) of granulosa cells from control and aminophylline (Ami) groups. **C**, Statistical validation of OPLS-DA by 7-fold cross validation and 200 × response permutation testing of metabolites. **D-E**, Volcano plot and metabolome data displayed differential metabolites (**D**) and the levels of phospholipid metabolites (**E**) in granulosa cells from control and aminophylline groups. **F-G**, Heatmap (**F**) and volcano plot (**G**) showed the differentially expressed genes in oocytes from control and aminophylline groups. **H**, KEGG analysis of the differentially expressed genes in oocytes from control and aminophylline groups. **I**, Heatmap showed the differences in the expression of a set of transcripts involving different processes between control and aminophylline groups. Bars indicate the mean ± SD. *P < 0.05, **P < 0.01, and ***P < 0.001. Two-tailed unpaired t-test was used to assess statistical significance.

We also studied the effects of aminophylline on the metabolome profiles of granulosa cells in naturally aged mice. Principal component analysis (PCA) and orthogonal partial least squares-discriminant analysis (OPLS-DA) revealed that the six biological replicate samples in each group clustered together but were distinctly separated between the aminophylline and control groups (Figure 7A and 7B). The OPLS-DA model showed a low risk of overfitting for that the green Q2 values to the left were all lower than the original points to the right (Figure 7C). In addition, volcano plot and heatmap analyses revealed significant alterations in the granulosa cell metabolome profiles between the aminophylline and the control groups (110 upregulated metabolites and 68 downregulated metabolites) (Figure 7D, Figure S10A). Kyoto Encyclopedia of Genes and Genomes (KEGG) analysis showed that autophagy was the most altered signaling pathway (Figure S10C and S10D). Notably, phosphatidylethanolamine (PE), a multifunctional phospholipid and an upregulated metabolite induced by aminophylline, was present in autophagy pathway (Figure S10B). Also, the analysis of metabolome data showed that the levels of phosphatidic acid (PA), phosphatidylcholine (PC), phosphatidylglycerol (PG), and phosphatidylserine (PS) were significantly increased in granulosa cells of aminophylline group compared with control (Figure 7E).

We further studied the effects of aminophylline on the oocyte transcriptome in naturally aged mice. Both the heatmap and volcano plot showed that the oocyte transcriptome was substantially altered in aminophylline group compared with control (459 upregulated transcripts and 589 downregulated transcripts) (Figure 7F and 7G). In KEGG analysis, the upregulated genes were associated with mTOR, HIF-1, and MAPK pathways, while the downregulated genes were associated with Hippo pathway and apoptosis (Figure 7H and 7I). Therefore, the improvement of oocyte quality induced by aminophylline may be mediated by increasing the levels of phospholipid metabolites in granulosa cells and altering regulatory pathways in oocytes.

### Theophylline derivatives promote human primordial follicle activation

**Figure 8:**
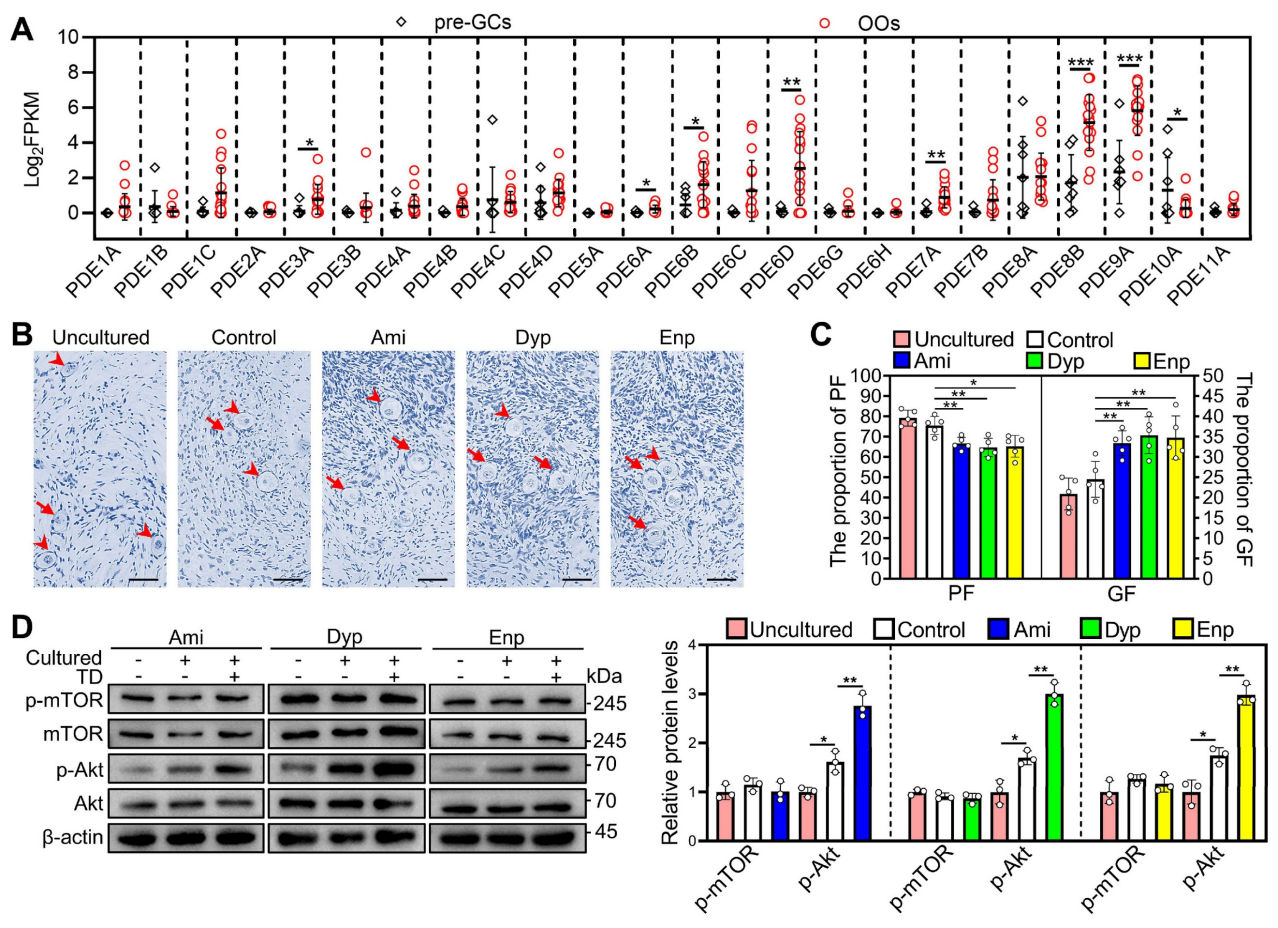
** Effects of theophylline derivatives on human primordial follicle activation *in vitro*.** The fragments of human ovarian tissue were directly collected (uncultured) or cultured in medium alone (control) or with 50 μM aminophylline (Ami), 160 μM dyphylline (Dyp), or 10 μM enprofylline (Enp) for 4 days, followed by cultured in medium alone for additional 2 days. The fragments were collected after 4 (**D**) or 6 days (**B-C**). **A**, The PDE subtype expression levels in pre-granulosa cells (n = 8 follicles) and oocytes (n = 17 follicles) of human primordial follicles. pre-GCs, pre-granulosa cells; OOs, oocytes. **B-C**, The comparison of human ovarian tissue fragmented morphology (**B**) and primordial and growing follicle (**C**) proportion across various groups. The human ovarian sections were hematoxylin-stained. **D**, The comparison of p-mTOR and p-Akt protein levels across various groups. The representative images were displayed. Arrowheads, primordial follicles; arrows, growing follicle. Scale bars, 50 µm. In each experiment, n ≥ 3 biological replicates. Bars indicate the mean ± SD. *P < 0.05 and **P < 0.01. Two-tailed unpaired t-test was used to assess statistical significance.

We used the published RNA-seq data (GEO: GSE107746) to analyze phosphodiesterase subtypes expression in human primordial follicles 36. *PDE3A*, *PDE6A*, *PDE6B*, *PDE6D*, *PDE7A*, *PDE8B*, and *PDE9A* mRNAs were expressed mainly in the oocytes (Figure 8A). Therefore, we further investigated the impact of theophylline derivatives on the activation of human primordial follicles by culturing human ovarian fragments. Compared with control, aminophylline, dyphylline, and enprofylline significantly increased p-Akt levels and growing follicle proportion after treatment for 4 and 6 days, respectively (Figure 8B-D). The results suggest that the theophylline derivatives activate PI3K/Akt pathway possibly by inhibiting oocyte PDE activity and then promote human primordial follicle activation. The p-Akt levels and growing follicle proportion were increased in control group compared with that in uncultured group (Figure 8B-D), suggesting the onset of follicle growth during the culture 37.

## Discussion

Cyclic AMP is crucial for maintaining the balance between primordial follicle dormancy and activation 25. In the present study, *Pde2a*, *Pde3a*, *Pde3b*, *Pde6d*, and *Pde8a* were expressed mainly in mouse primordial follicle oocytes. The specific PDE inhibitors nimodipine, EHNA, and zaprinast and the nonspecific PDE inhibitors theophylline derivatives activated mouse and/or human primordial follicles. The mechanism involves the accumulation of cAMP levels and the activation of the PI3K/Akt pathway in oocytes. Oral aminophylline increased oocyte quantity by promoting primordial follicle activation and ameliorating infertility in naturally aged mice.

PDE3A is expressed mainly in mouse primordial follicle oocytes, and the inhibition of PDE3A by milrinone leads to primordial follicle activation 25. However, the knockout of *Pde3a* showed no impact on mouse primordial follicle activation 26. In this study, *Pde2a*, *Pde3a*, *Pde3b*, *Pde6d*, and *Pde8a* were also expressed mainly in mouse primordial follicle oocytes, as well as *Pde1a* and* Pde2a* levels in ovaries were decreased during primordial follicle activation. The inhibition of PDE1A, PDE2A, and PDE5A/PDE6D leads to mouse primordial follicles activation. This suggests that all these PDE subtypes play a compensatory role with each other in maintaining primordial follicle dormancy.

In neonatal mouse ovaries, *Adcy2*, *Adcy3*, and *Adcy6* are expressed mainly in pre-granulosa cells 38. *Pde2a*, *Pde3a*, *Pde3b*, *Pde6d*, and *Pde8a* were expressed mainly in primordial follicle oocytes. The inhibition of PDE activity by PDEI and theophylline derivatives increased cAMP levels and then activated PI3K/Akt pathway in oocytes, consistent with previous findings that cAMP increases PI3K/Akt activity in various tissues 18, 19. In addition, the blockade of gap junctions using CBX increased cAMP levels and then activated pre-granulosa cell glycolysis-dependent mTOR pathway. Consistent with previous studies that cAMP stimulates glycolysis and the enhanced glycolysis activates mTOR pathway in various cells 2, 16, 17. Thus, pre-granulosa cell-produced cAMP enters oocytes through gap junctions, while PDEs in oocytes maintain mouse primordial follicle dormancy by hydrolysing cAMP.

In the aged mice, the oocytes exhibit a decreased ratio of high ΔΨm/low ΔΨm and increased ROS levels, the sign of decreased oocyte quality 15, 39. The oral administration of aminophylline improved oocyte quality by increasing the ratio of high ΔΨm to low ΔΨm and reducing ROS levels. The activation of cAMP/PKA signaling decreased cellular ROS levels in human umbilical vein endothelial cells 40, and alleviated β-amyloid (Aβ)-induced energy failure, ΔΨm collapse, and cell toxicity in astrocytes 41. Oral administration of aminophylline also upregulated the genes related to mTOR, HIF-1, and MAPK pathways, and downregulated the genes related to Hippo pathway and apoptosis. The PI3K/Akt/mTOR signaling pathway is beneficial in reducing oxidative stress and inhibiting cell apoptosis 42. MAPK, an essential downstream kinase of EGFR signaling, could promote mitochondrial biogenesis, cell growth and division, and upregulate β-oxidation to increase mitochondrial membrane potential 43. HIF-1 could promote mitophagy, remove the damaged or harmful mitochondria, and prevent the continued production of ROS 44. The decrease of the Hippo pathway could mitigate oxidative stress 45. Thus, these changed pathways may improve oocyte quality by enhancing mitochondrial function in aminophylline treatment group. In growing follicles, PDE1A, PDE4D, and PDE5A are also expressed in granulosa cells 46. Aminophylline promoted the production of phospholipid metabolites PE, PA, PC, PG, and PS in granulosa cells possibly by increasing cAMP levels. Both PS and PE are important components of cellular membranes. PE also has many cellular functions, including oxidative phosphorylation, mitochondrial biogenesis and autophagy 47, 48. Growth factor-stimulated phospholipase D (PLD) could catalyze PC hydrolysis to PA that activates the mTOR-HIF-1-VEGF pathway for cell proliferation and survival 49. PG is a critical component of the inner mitochondrial membrane and is also involved in the biosynthesis of cardiolipin that plays a crucial role in maintaining the integrity and function of the mitochondrial membrane 50. Therefore, the increased levels of these phospholipid metabolites may enhance the function of granulosa cells in aging mice, thereby promoting the growth and development of oocytes.

The remaining primordial follicles of elderly women and patients with POI are difficult to be activated under the physiological condition. A limited number of young POI patients have successfully used IVA to obtain their own offspring, but this process is associated with risks of carcinogenesis and offspring defects 12-14. In the present study, the theophylline derivatives promoted mouse and human primordial follicle activation *in vitro*, and oral theophylline derivatives also promoted mouse primordial follicle activation without obvious side effects on the vital organs. Furthermore, oral aminophylline ameliorated infertility in naturally aged mice by enhancing oocyte quality and quantity. In addition, the oral doses of aminophylline, dyphylline, and enprofylline for mice were 73.41, 139.84, and 6.77 mg/kg/d, respectively (equal to 5.95, 11.33, and 0.55 mg/kg/d for humans). Aminophylline at 5.00-10.00 mg/kg/d and enprofylline at 5.00-16.67 mg/kg/d have been utilized in the treatment of human asthma 51, 52. Importantly, these theophylline derivatives also promoted human primordial follicle activation, and oral aminophylline with 300 mg/d for 4-6 months obviously increased anti-Müllerian hormone (AMH) levels in POI patients (data not shown). Thus, these oral drugs derived from theophylline have the potential to treat POI patients and aged women. There are about 30 synthetic theophylline derivatives, and each has different effective concentrations and curative effects. Further experiments are needed to screen more effective theophylline derivatives and/or their combinations to meet the diverse requirements of POI patients.

**Figure 9:**
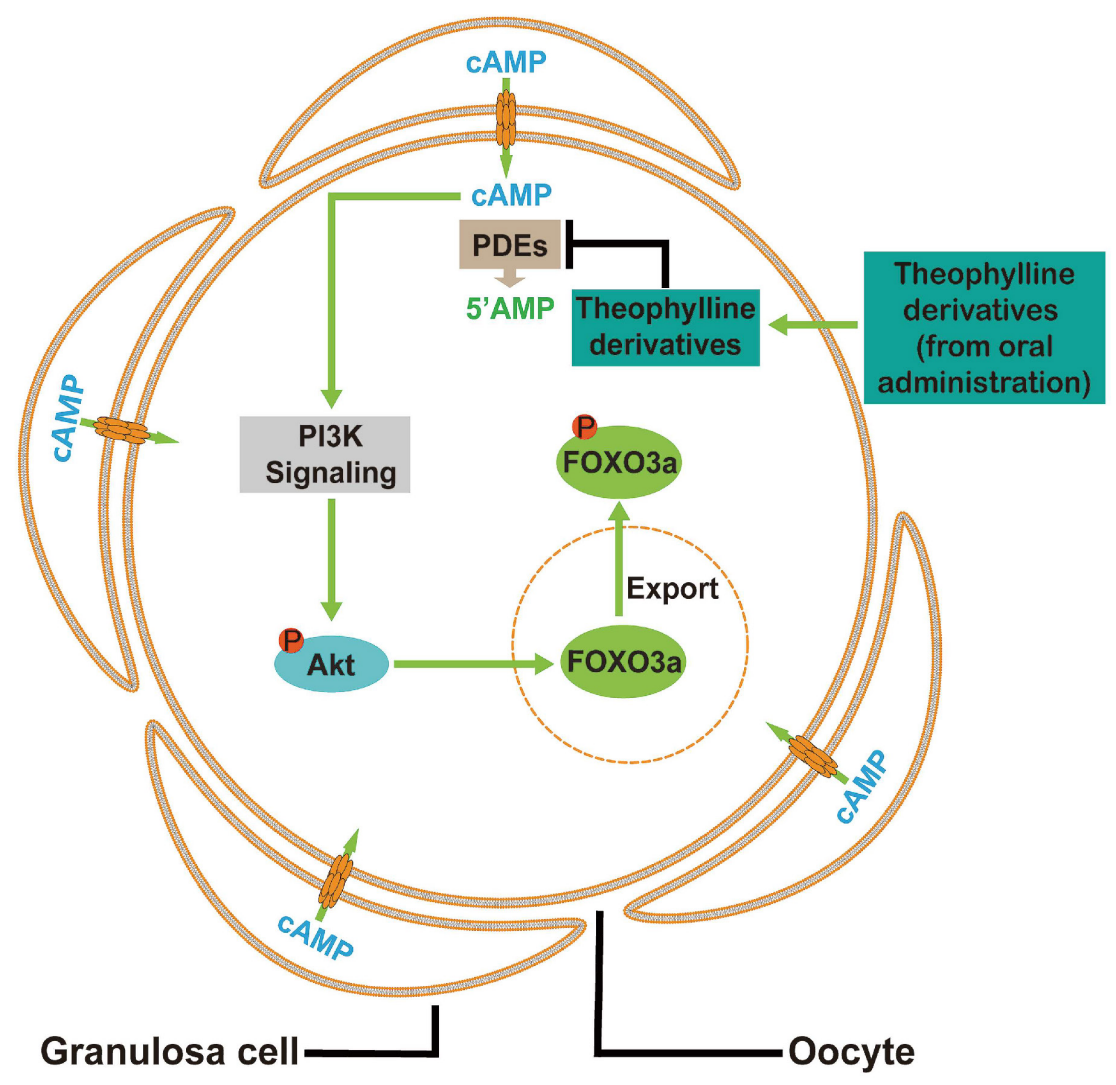
** A model depicting theophylline derivatives-mediated primordial follicle activation.** Cyclic AMP produced by pre-granulosa cells enters oocytes through gap junctions, which is degraded by PDEs to maintain the dormant state of primordial follicles. Theophylline derivatives, as PDE inhibitors, activate the PI3K/Akt signaling pathway in oocytes by the accumulation of cAMP, leading to the activation of primordial follicles.

In conclusion, cAMP from pre-granulosa cells is degraded by oocyte PDEs to maintain the dormancy of primordial follicles. The theophylline derivatives activated mouse and human primordial follicles via the cAMP-PI3K/Akt pathway in oocytes (Figure 9). Oral aminophylline also ameliorated infertility in naturally aging mice by increasing ovulated oocyte quality and quantity. Because of their effectiveness and safety, these oral drugs derived from theophylline could be a promising clinical strategy for ameliorating infertility in various groups of POI patients and aged women.

## Materials and methods

### Animals and chemicals

The ICR mice were obtained from the Guangdong Medical Laboratory Animal Center (Guangzhou, China) and were housed in South China University of Technology with a dark-light cycle of 12/12-hour at constant temperature. The newborn mice were acquired through adult female and male mouse breeding, with the day of birth defined as 0.5 dpp. These newborn female mice were utilized for ovary culture and intraperitoneal injection. The female mice at 21 days and 14 months were utilized for the oral administration trials. All reagents were obtained from Sigma-Aldrich (St. Louis, MO, USA) unless stated otherwise.

### Separation of ovarian somatic cells and oocytes

The 4 dpp mouse ovaries were isolated and then digested with 0.05% trypsin (Thermo Fisher Scientific, Waltham, MA, USA) at 37 °C for 10 minutes. The digestion was stopped by adding 10% fetal bovine serum (FBS, Thermo Fisher Scientific). Under a stereomicroscope, all oocytes were collected and then transferred to phosphate buffered saline (PBS) containing 0.2% bovine serum albumin (BSA) for 3-5 times until few somatic cells were observed. Oocytes and somatic cells were collected by centrifugation and then were stored at -80 °C for RNA analysis. The majority of ovarian somatic cells are pre-granulosa cells.

### RNA extraction and quantitative real-time PCR (qRT-PCR) analysis

Total RNA was extracted from mouse ovaries using the TRIzol reagent (Thermo Fisher Scientific). Subsequently, the RNA concentration and quality were assessed using a NanoDrop™ One Spectrophotometer (Thermo Fisher Scientific). cDNA was synthesized from one microgram of RNA using the GoScript™ Reverse Transcription System (Promega, Madison, WI, USA). For RNA extracted from ovarian somatic cells or oocytes using the RNeasy® Micro Kit (Qiagen, Valencia, CA, USA), cDNA was synthesized using the QuantiTect® Reverse Transcription Kit (Qiagen). qRT-PCR was performed using the SuperMix (Trans, Beijing, China) on a PCR machine (Roche, Basel, Switzerland). The internal control was Ribosomal protein L19 (*Rpl19*). BGI Genomics (BGI-Tech, Shenzhen, China) synthesized all required primers and all primer sequences were shown in Table S2.

### Mouse ovary culture

Mouse ovary culture was carried out as the previous study 15. Briefly, 3 dpp mouse ovaries were isolated and cultured in the medium alone or with nimodipine (0-100 μM), EHNA (0-5 μM), zaprinast (0-10 μM), BRL-50481 (0-50 μM), PF-04957325 (0-10 mM), CBX (0-15 μM), aminophylline (0-100 μM), dyphylline (0-240 μM), enprofylline (0-50 μM), LY294002 (10 μM), 2-DG (5 mM), and/or PI3K-IN-1 (25 μM). In some experiments, 3 dpp mouse ovaries were cultured in the medium alone or with 50 μM aminophylline for indicated time. The concentrations of LY294002, 2DG, and PI3K-IN-1 were referenced from published articles 2, 53, 54. For the stock solutions, EHNA, aminophylline, and dyphylline were prepared in physiological saline, and the others were prepared in dimethyl sulfoxide (DMSO). The culture medium was DMEM/F12 (Thermo Fisher Scientific, Waltham, MA, USA) containing 100 UI/ml penicillin-streptomycin, 3 mg/ml BSA, and 1% insulin-transferrin-selenium (ITS), and was refreshed every two days. The incubation condition was 5% CO_2_, 37 °C, and saturated humidity. The ovaries were utilized for follicle counting, immunofluorescence staining, cAMP and cGMP level analysis, as well as mRNA and protein assays.

### Histological analysis and follicle counting

Ovarian tissues and certain mouse vital organ samples were fixed, dehydrated, embedded, and sectioned serially as reported before 15. After dewaxing, the sections were stained with hematoxylin (Solarbio, Beijing, China). The primordial and growing follicles of neonatal mouse ovaries were counted in every fifth section and in consecutive sections, respectively. In each ovary, the total primordial follicle number was mean follicle number per section × total section number. The primordial and growing follicles of other ovarian tissues were counted through consecutive sections. Only follicles containing a clearly visible nucleus of oocyte were used for counting.

### Immunofluorescence

Immunofluorescence was carried out as the previous study 15. Briefly, mouse ovary sections were dried, deparaffinized, and microwaved for antigen retrieval. Following a 1-hour block with 10% donkey serum, the sections were overnight incubation at 4 °C with primary antibodies (Table S1), followed by a 1-hour incubation with secondary antibodies conjugated to Alexa Fluor 488 or 555 (Thermo Fisher Scientific). After a 5-minute stain with DAPI, the sections were treated with anti-fluorescence quenching agents (Ruitaibio, Beijing, China), and imaged using a confocal microscope. A primordial follicle was considered positive if more than one pre-granulosa cell exhibited staining. Zeiss Zen 3.0 software was used to measure fluorescence intensity, and relative fluorescence intensity was determined by calculating the ratio of cell fluorescence intensity to that of the background. The mean value of the five largest sections in each ovary was considered one independent repetition. For spindle staining, oocytes from naturally aged mice were fixed for 30 minutes in 4% paraformaldehyde (PFA), permeabilized for 20 minutes in 0.5% Triton X-100 in PBS, and blocked for 1 hour with 10% donkey serum. Then, they were stained with anti-alpha tubulin antibody (1:300) coupled with Alexa Fluor 488 for 3 hours. Following a 10-minute DAPI staining, oocytes were mounted on slides with anti-fluorescence quenching agents and imaged using a confocal microscope.

### BrdU incorporation assay

Ovary samples from 3 dpp mice were treated with different drugs for 2 days, and then incubated with BrdU (10 μM) for further 2 hours in drug-free medium. Serial sections were obtained from the ovaries as previously described. The sections were retrieved, blocked and then subjected to an overnight incubation at 4 ℃ with anti-BrdU antibody, followed by a 3 hours incubation with Alexa Fluor 488-conjugated donkey secondary antibody. The mean number of BrdU-positive cells in the five largest sections of each ovary was considered as one independent repetition.

### Western blotting analysis

The ovaries from each group were lysed by radio immunoprecipitation assay lysis buffer (Beyotime, Beijing, China) containing 1 mM phenylmethylsulfonyl fluoride (PMSF, Beyotime). The protein concentration was assessed using the bicinchoninic acid (BCA) assay (Beyotime). Subsequently, the same amounts of protein (15 μg) mixed with sodium dodecyl sulfate (SDS) buffer (Cwbio, Beijing, China) were loaded into a 5% stacking and 10% separating gel. Electrophoresis was conducted at 60V for 40 minutes, followed by 100V for 60 minutes. After electrophoresis, the proteins were transferred to polyvinylidene difluoride (PVDF) membranes at 100V for 60 minutes. The membrane was blocked in 5% skim milk for 1 hour at room temperature and then incubated overnight at 4 °C with primary antibodies (Table S1). After washing with tris buffered saline tween (TBST), the membranes were incubated for 1 hour at room temperature with anti-mouse or anti-rabbit IgG secondary antibody (1:5000. ZSGB-BIO, Beijing, China). Following additional washing with TBST, protein bands on the membranes were visualized using chemiluminescent substrate (Thermo Fisher Scientific) and imaged using chemiluminescent imaging system (Tanon, Shanghai, China). Band density was quantified using ImageJ software (NIH Image, Bethesda, MD, USA), and protein expression levels were normalized to β-actin.

### Measurement of cAMP and cGMP levels

The ovaries collected from different groups were weighed (g) and added to 9 times the volume of PBS (ml) corresponding to the weight of the ovaries, and then crushed into homogenates using an automatic sample grinder (Jingxin, Shanghai, China). These homogenates were repeatedly freeze-thawed to further lyse the cells, followed by centrifugation at 5000 g for 10 min to collect the supernatants. The protein concentration in the supernatants was detected by BCA assay (Beyotime), while cAMP and cGMP concentrations were measured using the ELISA kits purchased from RUIXIN Biotech (Quanzhou, China).

### Drug injection and oral administration

The drug doses for a single injection (mg/kg) were calculated according to their optimal concentrations (mg/L) used in the culture. 3 dpp female mice were intraperitoneally injected twice a day with PDEI (4.18 mg/kg nimodipine, 0.79 mg/kg EHNA, and 1.36 mg/kg zaprinast), 21.02 mg/kg aminophylline, 40.68 mg/kg dyphylline, 1.94 mg/kg enprofylline or physiological saline for two consecutive days. The ovaries were utilized for immunofluorescence and protein analysis and for follicle counting at 12 hours and 2 days post-injection, respectively.

In order to administer drugs via drinking water, we deprived adolescent mice of water for 6 hours (from 9:00 to 15:00) and then provided them with drinking water containing theophylline derivatives for 30 minutes. Studies have shown that depriving mice of water for 6 hours during the day has no side effect 55. According to the preliminary experiments, the average drinking water was 0.05 L/kg under this condition. The drug concentration in drinking water (2, 6.5, and 0.4 mM for aminophylline, dyphylline, and enprofylline, respectively) was calculated based on the average drinking water (~ 0.05 L/kg), in which the daily intake dose of the drug from water is the same as the daily injection dose of the drug. Additionally, we set up 0.5, 1, and 2 times drug concentrations in the drinking water, and found that the most effective drug concentrations for the activation of primordial follicles were 2 times drug concentrations (4, 13, and 0.8 mM for aminophylline, dyphylline, and enprofylline, respectively). We recorded the actual drug intake by drinking water and body weight of the mice every day and calculated the average drug intake doses during the 7-day treatment. Similarly, the 14-month-old naturally aged mice were administered either water alone or water containing 4 mM aminophylline for one week. The oocyte quality analysis, follicle counting, and fertility testing were studied three weeks later.

### Ovulation analysis and fertility testing

The naturally aged female mice with or without aminophylline treatment were induced to ovulate through intraperitoneal injection of equine chorionic gonadotropin (eCG, 5 IU). After 48 hours, they were given human chorionic gonadotrophin (hCG, 5 IU) through intraperitoneal injection. The cumulus-oocyte complexes were collected 14 hours post-hCG, and then the cumulus cells were removed by 0.1% hyaluronidase. Oocyte quantity and quality were analyzed by assessing ROS, ΔΨm, and spindle staining. To examine fertility, the treated and untreated naturally aged female mice were mated with fertile male mice, and the number of newborn mice was recorded weekly.

### ΔΨm and ROS measurements

The oocyte ΔΨm and ROS levels were detected following the manufacturer's instructions (Beyotime, Beijing, China). To detect ΔΨm, the oocytes were incubated with JC-1 in buffer solution for 30 minutes. To detect ROS, the oocytes were incubated with 2',7'-dichlorodihydrofluorescein diacetate (10 μM) in M2 medium for 30 minutes. All the oocytes were imaged using a confocal microscopy (Carl Zeiss, Oberkochen, Germany).

### Transcriptome sequencing and metabolomic analysis

Ovaries were obtained from naturally aged mice that were intraperitoneally injected with eCG. Oocytes were collected by puncturing the large antral follicles under a stereoscopic microscope and were kept in sample buffer for RNA extraction and transcriptome sequencing at OE Biotech Co., Ltd. (Shanghai, China). Granulosa cells were collected from the large antral follicles for non-targeted metabolomic analysis at OE Biotech Co., Ltd.

### Human ovarian tissue culture

Small ovarian cortical biopsy specimens (adjacent nonpathologic tissue) were obtained from seven women aged between 23 and 42 years (mean age 31.1 ± 7.8 years) at Zhongshan City People's Hospital (Zhongshan, Guangdong, China) and The Sixth Affiliated Hospital of Sun Yat-sen University (Guangzhou, Guangdong, China). Upon arrival at the laboratory, the ovarian tissues were cut into 1 mm³ fragments. Some fresh fragments (uncultured group) were used for protein detection or follicle counting. The remaining fragments were equally and randomly distributed into different culture groups. The human ovarian tissues were cultured either alone or in the presence of 50 μM aminophylline, 160 μM dyphylline, or 10 μM enprofylline for 4 and 6 days for protein examination and follicle counting, respectively.

### Statistical analysis

All data were presented as mean ± SD and analyzed using two-tailed unpaired Student's t tests. The graphical representations of all data were created using GraphPad Prism software (La Jolla, CA, USA).

## Supplementary Material

Supplementary figures and tables.

## Abbreviations

8br-cGMP: 8-bromo-cGMP
ADCYs: adenylate cyclases
Akt: protein kinase B
Aldoa: aldolase A
AMH: anti-Müllerian hormone
Ami: aminophylline
ART: assisted reproductive technology
Bax: B-cell lymphoma 2-associated X
BCA: bicinchoninic acid
BrdU: bromodeoxyuridine
Bcl-2: B-cell lymphoma 2
cAMP: cyclic adenosine monophosphate
CBX: carbenoxolone
cGMP: cyclic guanosine monophosphate
CL: corpus luteum
COPD: chronic obstructive pulmonary disease
dbcAMP: dibutyryl cAMP
DDX4: DEAD-box helicase 4
DMEM: Dulbecco's modified Eagle's medium
DMSO: dimethyl sulfoxide
dpp: days postpartum
Dyp: dyphylline
EA: early antral follicle
eCG: equine chorionic gonadotropin
Enp: enprofylline
Eno1: enolase 1
EPAC: exchange protein activated by cAMP
FOXO3a: forkhead box O3a
GC: granulosa cell
GDF9: growth differentiation factor 9
GF: growing follicle
Glut4: glucose transporters type 4
hCG: human chorionic gonadotrophin
HCN: hyperpolarization-activated cyclic nucleotide-gated
Hk1: hexokinase 1
ITS: insulin-transferrin-selenium
IVA: *In vitro* activation
KEGG: Kyoto Encyclopedia of Genes and Genomes
KITL: proto-oncogenic receptor tyrosine kinase ligand
LA: late antral follicle
Ldhb: lactate dehydrogenase B
LY: LY294002
mTOR: mammalian target of rapamycin
OO: oocyte
OPLS-DA: orthogonal partial least squares-discriminant analysis
PA: phosphatidic acid
PBS: phosphate buffered saline
PC: phosphatidylcholine
PCA: Principal-component analysis
PCNA: proliferating cell nuclear antigen
PDE: phosphodiesterase
PE: phosphatidylethanolamine
PF: primordial follicle
PFA: paraformaldehyde
Pfkl: phosphofructokinase liver type
PG: phosphatidylglycerol
PI3K: phosphatidylinositol 3-kinase
PKA: protein kinase A
Pkm2: pyruvate kinase M2
PMSF: phenylmethylsulfonyl fluoride
POI: premature ovarian insufficiency
pre-GCs: pre-granulosa cells
PrF: primary follicle
PS: phosphatidylserine
PVDF: polyvinylidene difluoride
qRT-PCR: Quantitative real-time PCR
ROS: reactive oxygen species
Rpl19: Ribosomal protein L19
SDS: sodium dodecyl sulfate
SF: secondary follicle
TBST: tris-buffered saline tween
TD: theophylline derivatives
Tpi: triosephosphate isomerase
ZP3: zona pellucida glycoprotein 3
ΔΨm: mitochondrial membrane potential
