# Supplementary material for: Theophylline derivatives promote primordial follicle activation via cAMP-PI3K/Akt pathway and ameliorate fertility deficits in naturally aged mice
Source: Int J Biol Sci. 2024 Sep 30;20(13):5312–29. doi: 10.7150/ijbs.99936 (PMC11489179; doi:10.7150/ijbs.99936)
Supplement: Supplementary file 1 — Supplementary figures and tables. [file ijbsv20p5312s1.pdf]

Supplementary Material

Theophylline derivatives promote primordial follicle activation via cAMP-PI3K/Akt pathway and ameliorate fertility deficits in naturally aged mice

Wenbo Zhang[1#], Longwei Gao[1#], Xiaodan Zhang[1], Yashuang Weng[1], Yan Du[1], Yan-Li Sun[1], Hongwei Wei[1], Tiantian Hao[1], Yuezhou Chen[3*], Xiaoyan Liang[2*], Meijia Zhang[1*]

[1]The Innovation Centre of Ministry of Education for Development and Diseases, The Second Affiliated Hospital, School of Medicine, South China University of Technology, Guangzhou, Guangdong, 510006, China.

[2]Reproductive Medicine Research Center, The Sixth Affiliated Hospital of Sun Yat-sen University, Guangzhou, Guangdong, 510655, China.

[3]Reproductive Medicine Center, Zhongshan City People's Hospital, Zhongshan, Guangdong, 528403, China.

[#]These authors contributed equally: Wenbo Zhang, Longwei Gao.

[*]Corresponding authors: Meijia Zhang (zhangmeijia@scut.edu.cn); Xiaoyan Liang (liangxy2@mail.sysu.edu.cn); Yuezhou Chen (yzchen415@zju.edu.cn).

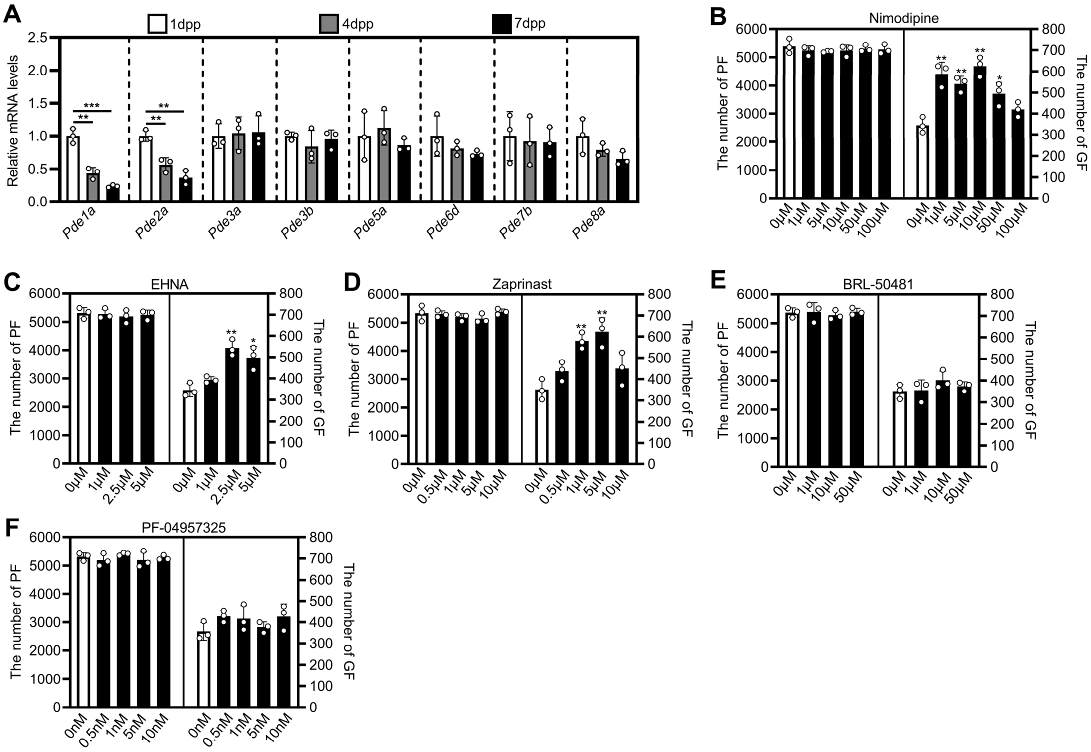

Figure S1. Effects of specific phosphodiesterase inhibitors on mouse primordial follicle activation *in vitro*. 1, 4, and 7 dpp mouse ovaries were collected for qRT–PCR analysis (**A**). 3 dpp mouse ovaries were cultured in medium alone or with nimodipine, EHNA, zaprinast, BRL-50481, or PF-04957325 in different concentrations for 4 days (**B-F**). **A**, The comparison of highly expressed phosphodiesterase subtype mRNA levels in 1,4 and 7dpp mouse ovaries (n = 3 biological replicates). ***P* < 0.01, ****P* < 0.001 vs. 1 dpp group. **B-F**, The comparison of primordial and growing follicle number across various groups. PF, primordial follicle; GF, growing follicle. In each experiment, n = 3 biological replicates. Bars indicate the mean ± SD. **P* < 0.05, ***P* < 0.01 and ****P* < 0.001. Two-tailed unpaired t-test was used to assess statistical significance.

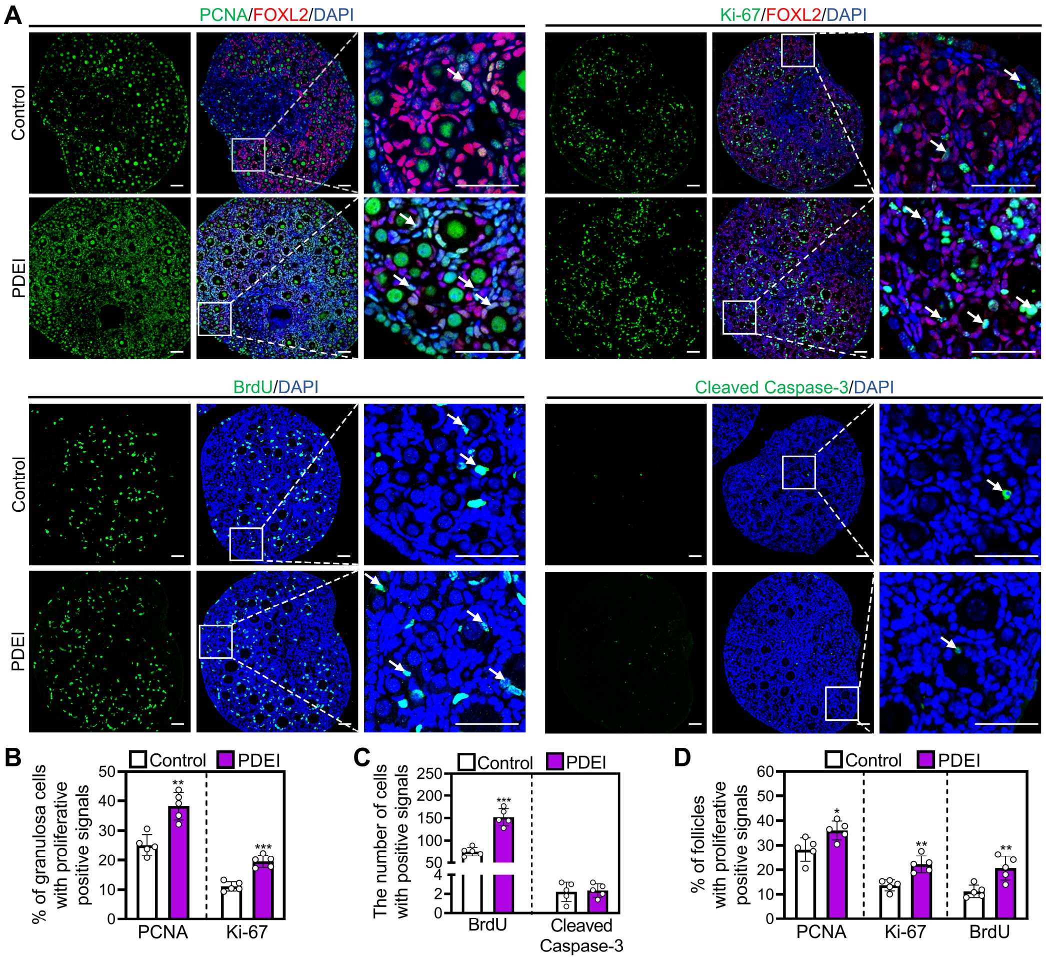

Figure S2. Effects of PDEI on granulosa cell proliferation *in vitro*. 3 dpp mouse ovaries were cultured in medium alone or with PDEI (the combination of 10 μM nimodipine, 2.5 μM EHNA, and 5 μM zaprinast) for 2 days. **A,** PCNA, Ki-67, BrdU, and Cleaved Caspase-3 immunofluorescence stain (green) in control and PDEI group. FOXL2, red. **B-C**, The comparison of PCNA- and Ki-67-positive percentage in granulosa cells (**B**) and BrdU- and Cleaved Caspase-3-positive cell number (**C**) between control and PDEI groups. **D**, The comparison of PCNA-, Ki-67- and BrdU- positive percentage in primordial follicles between control and PDEI groups. The representative images were displayed. Scale bars, 50 μm. In each experiment, n = 5 biological replicates. Bars indicate the mean ± SD. *$P < 0.05$, **$P < 0.01$ and ***$P < 0.001$. Two-tailed unpaired t-test was used to assess statistical significance.

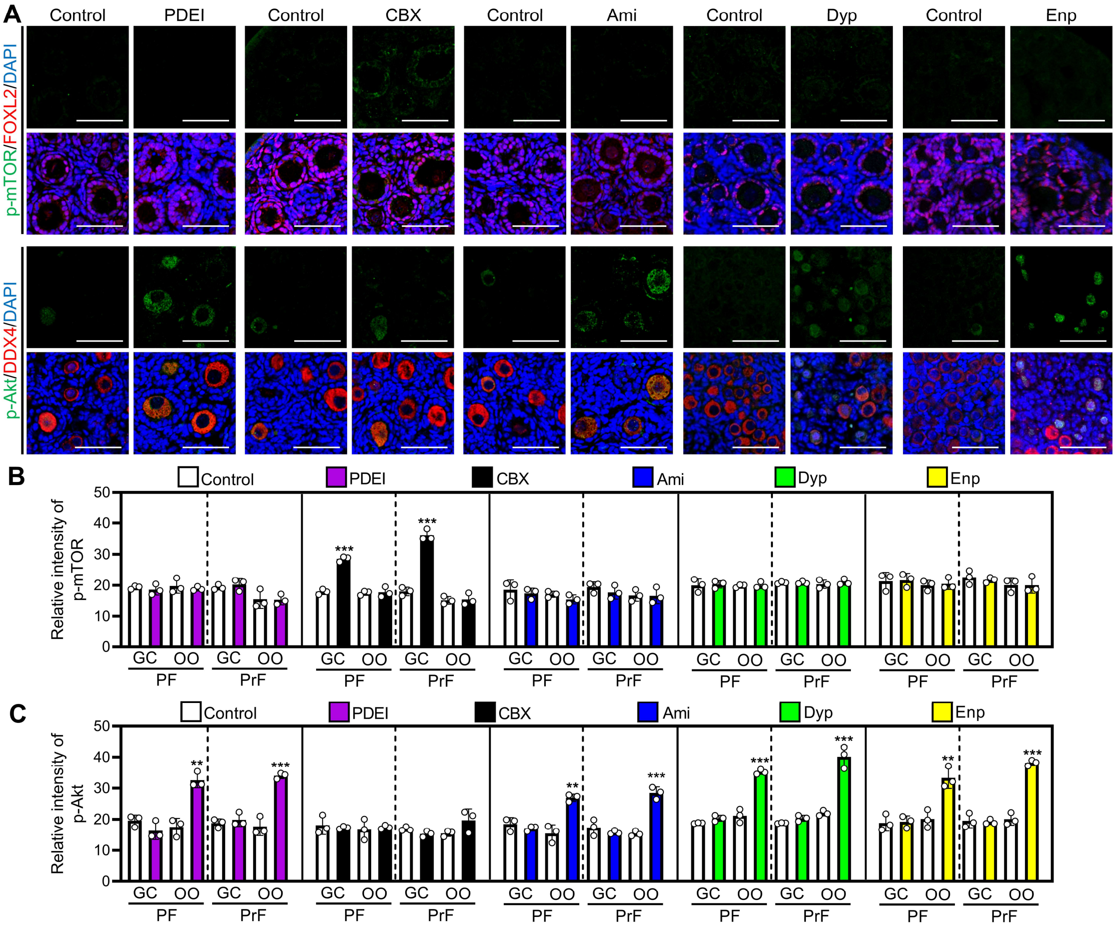

Figure S3. Effects of CBX, PDEI, and the theophylline derivatives on p-mTOR and p-Akt fluorescent intensities in cultured mouse ovaries. 3 dpp mouse ovaries were cultured in medium alone or with PDEI (the combination of 10 μM nimodipine, 2.5 μM EHNA, and 5 μM zaprinast), 10 μM CBX, 50 μM aminophylline (Ami), 160 μM dyphylline (Dyp), or 10 μM enprofylline (Enp) for 1 day. **A**, p-mTOR and p-Akt (green) immunofluorescence stain across various groups. FOXL2 or DDX4, red. **B-C**, The p-mTOR (**B**) and p-Akt (**C**) fluorescent intensities in granulosa cells and oocytes of primordial follicles and primary follicles. The representative images were displayed. GC, granulosa cell; OO, oocyte; PF, primordial follicle; PrF, primary follicle. Scale bars, 50 μm. In each experiment, n = 3 biological replicates. Bars indicate the mean ± SD. ***P* < 0.01 and ****P* < 0.001. Two-tailed unpaired t-test was used to assess statistical significance.

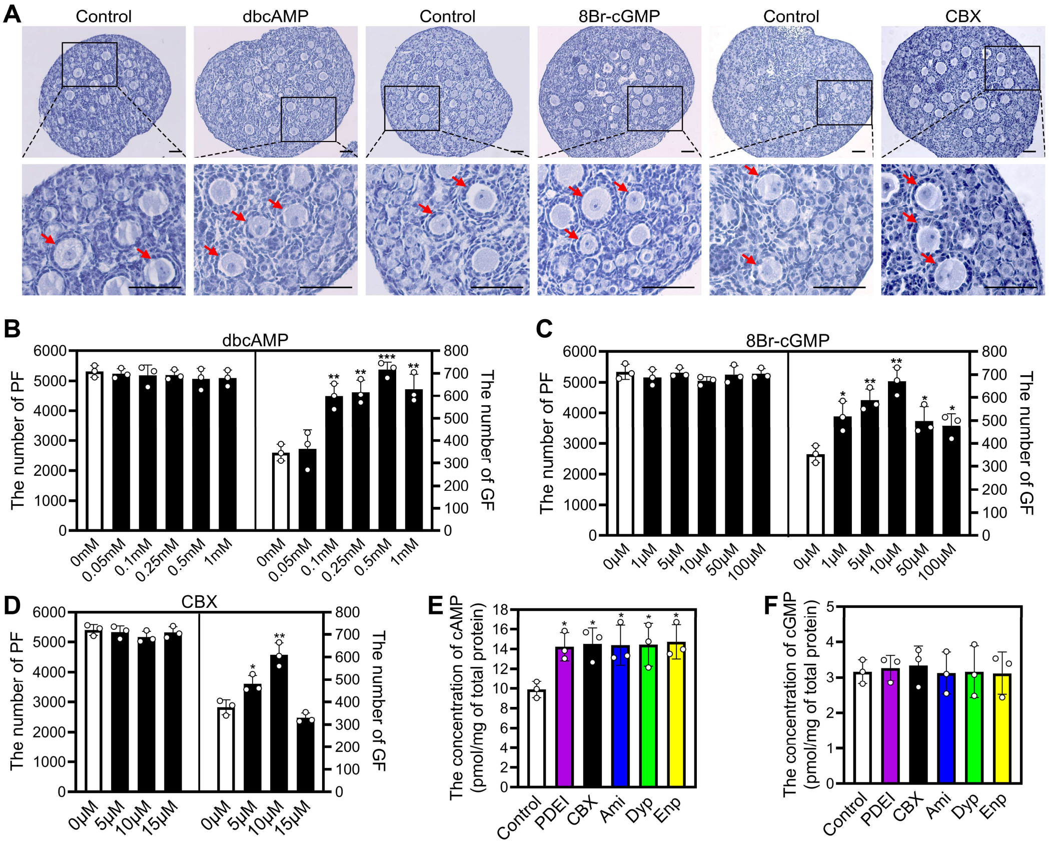

Figure S4. Effects of dbcAMP, 8Br-cGMP and CBX on mouse primordial follicle activation *in vitro*. 3 dpp mouse ovaries were cultured in medium alone or with dbcAMP, 8Br-cGMP, or CBX in different concentrations for 4 days (**A-D**), or cultured with PDEI (the combination of 10 μM nimodipine, 2.5 μM EHNA, and 5 μM zaprinast), 10 μM CBX, 50 μM aminophylline (Ami), 160 μM dyphylline (Dyp), or 10 μM enprofylline (Enp) for 1 day (**E-F**). **A-D**, The comparison of ovarian morphology (**A**) and primordial and growing follicle number (**B-D**) across various groups. The ovarian sections were hematoxylin-stained. **E-F**, The comparison of cAMP (**E**) and cGMP (**F**) concentration across various groups. The representative images were displayed. PF, primordial follicle; GF, growing follicle. Red arrows, growing follicles. In each experiment, n = 3 biological replicates. Bars indicate the mean ± SD. *$P < 0.05$, **$P < 0.01$ and ***$P < 0.001$. Two-tailed unpaired t-test was used to assess statistical significance.

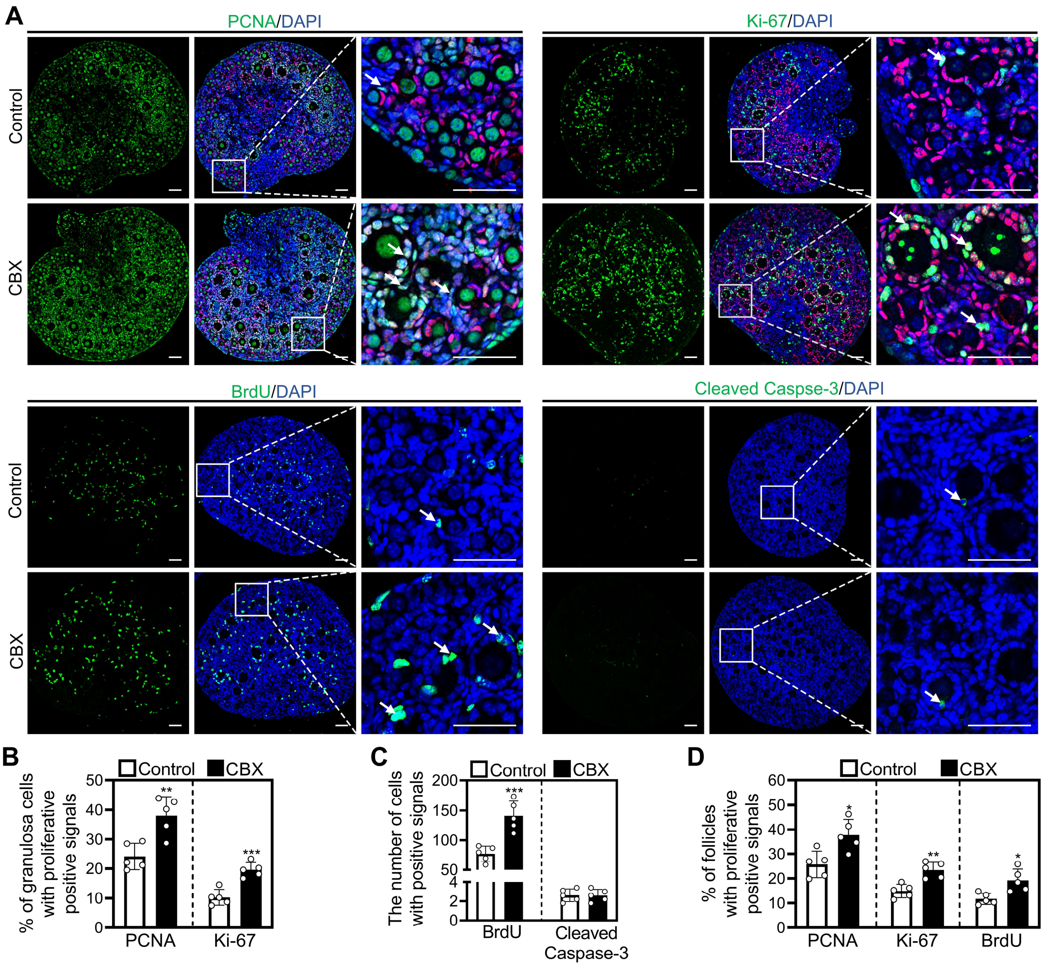

Figure S5. Effects of CBX on granulosa cell proliferation *in vitro*. 3 dpp mouse ovaries were cultured in medium alone or with 10 µM CBX for 2 days. **A**, PCNA, Ki-67, BrdU, and Cleaved Caspase-3 immunofluorescence stain (green) in control and CBX groups. FOXL2, red. **B-C**, The comparison of PCNA- and Ki-67-positive percentage in granulosa cells (**B**) and BrdU- and Cleaved Caspase-3-positive cell number (**C**) between control and CBX groups. **D**, The comparison of PCNA-, Ki-67- and BrdU- positive percentage in primordial follicles between control and CBX groups. The representative images were displayed. Scale bars, 50 µm. In each experiment, n = 5 biological replicates. Bars indicate the mean ± SD. *$P < 0.05$, **$P < 0.01$ and ***$P < 0.001$. Two-tailed unpaired t-test was used to assess statistical significance.

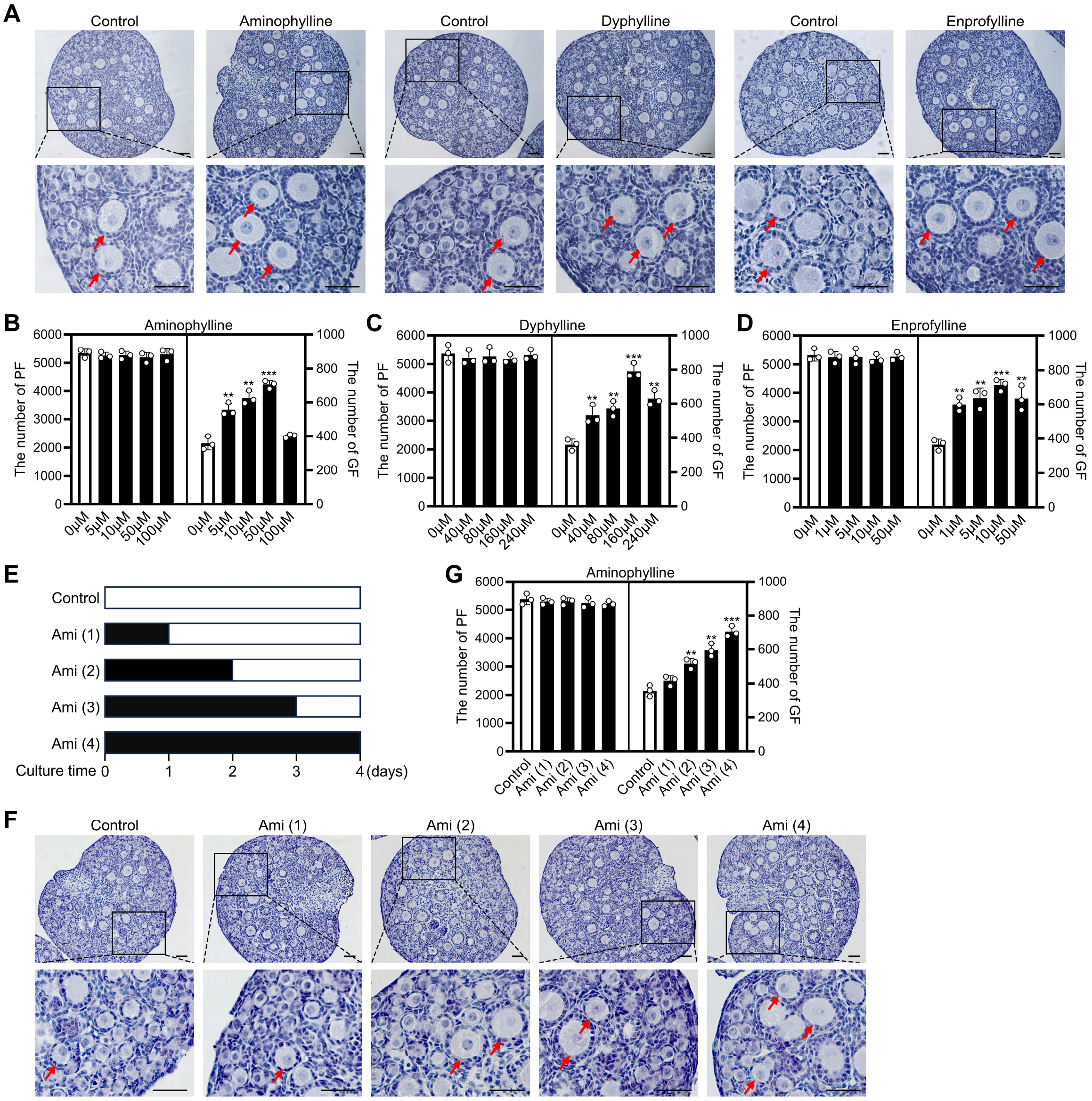

Figure S6. Effects of the theophylline derivatives on mouse primordial follicles activation *in vitro*. 3 dpp mouse ovaries were cultured in medium alone or with aminophylline, dyphylline, or enprofylline in different concentrations for 4 days (**A-D**) or cultured in medium alone or with aminophylline for indicated times (**E-G**). **A-D**, The comparison of ovarian morphology (**A**) and primordial and growing follicle number (**B-D**) across various groups. **E,** The timeline of the experiment was shown. 3 dpp mouse ovaries were cultured in medium alone or with aminophylline for indicated times and collected after 4 days of culture. **F-G**, The comparison of ovarian morphology (**F**) and primordial and growing follicle number (**G**) across various groups. Ami: aminophylline. The ovarian sections were hematoxylin-stained. Red arrows,

growing follicles. The representative images were displayed. Scale bars, 50 μm. PF, primordial follicle; GF, growing follicle. In each experiment, n = 3 biological replicates. Bars indicate the mean ± SD. $^{**}P < 0.01$ and $^{***}P < 0.001$. Two-tailed unpaired t-test was used to assess statistical significance.

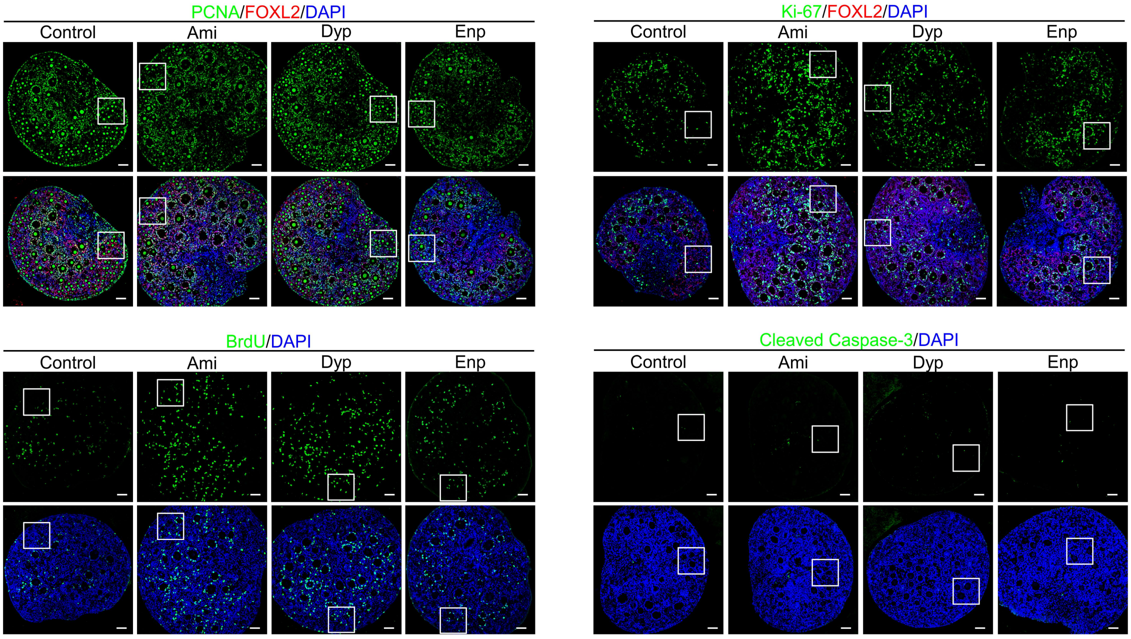

Figure S7. Effects of the theophylline derivatives on granulosa cell proliferation *in vitro* 3 dpp mouse ovaries were cultured in medium alone or with 50 μM aminophylline (Ami), 160 μM dyphylline (Dyp), or 10 μM enprofylline (Enp) for 2 days. PCNA, Ki-67, BrdU, and Cleaved Caspase-3 (green) immunofluorescence stain across various groups. FOXL2, red. Figure 3H showed enlarged view of the boxed areas. Scale bars, 50 μm.

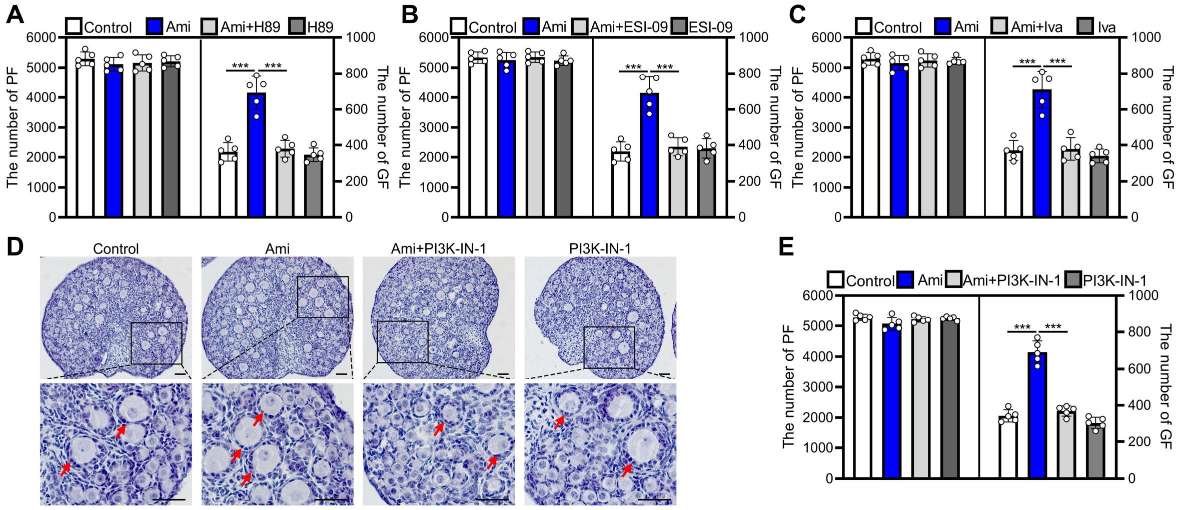

Figure S8. Effects of H89, ESI-09, ivabradine, and PI3K-IN-1 on aminophylline-induced mouse primordial follicle activation. 3 dpp mouse ovaries were cultured in medium alone, supplemented with 50 μM aminophylline (Ami), 5 μM H89, 5 nM ESI-09, 2.5 μM ivabradine (Iva), and/or 25 μM PI3K-IN-1 for 4 days. **A-C**, The comparison of primordial and growing follicle number across various groups. **D-E**, The comparison of ovarian morphology (**D**) and primordial and growing follicle number (**E**) across various groups. The ovarian sections were hematoxylin-stained. Red arrows, growing follicles. The representative images were presented. Scale bars, 50 μm. PF, primordial follicle; GF, growing follicle. In each experiment, n = 5 biological replicates. Bars indicate the mean ± SD. ***$P < 0.001$. Two-tailed unpaired t-test was used to assess statistical significance.

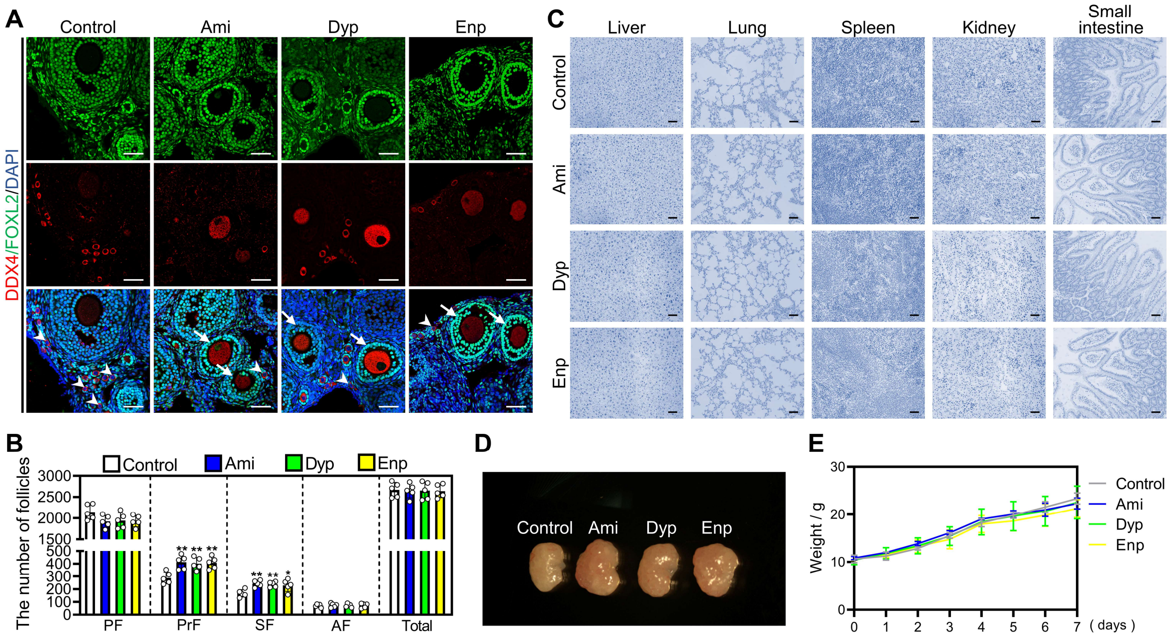

Figure S9. Effects of oral theophylline derivatives on adolescent mice. The adolescent mice were administered water either with or without 4 mM aminophylline (Ami), 13 mM dyphylline (Dyp), or 0.8 mM enprofylline (Enp) for one week, followed by the collection of ovaries and organs. **A-B**, The comparison of ovarian morphology (**A**) and follicle number at different stages (**B**) across various groups. DDX4, red; FOXL2, green. **C,** Morphological comparison of liver, spleen, kidney and small intestine. **D**, The adolescent mice ovarian morphology across various groups. **e**, The body weight change across various groups. The representative images were displayed. Arrowheads, primordial follicles; arrows, growing follicles. PF, primordial follicle; PrF, primary follicle; SF, secondary follicle; AF, antral follicle. Scale bars, 50 μm. In each experiment, n ≥ 3 for biological replicates. Bars indicate the mean ± SD. *P < 0.05 and **P < 0.01. Two-tailed unpaired t-test was used to assess statistical significance.

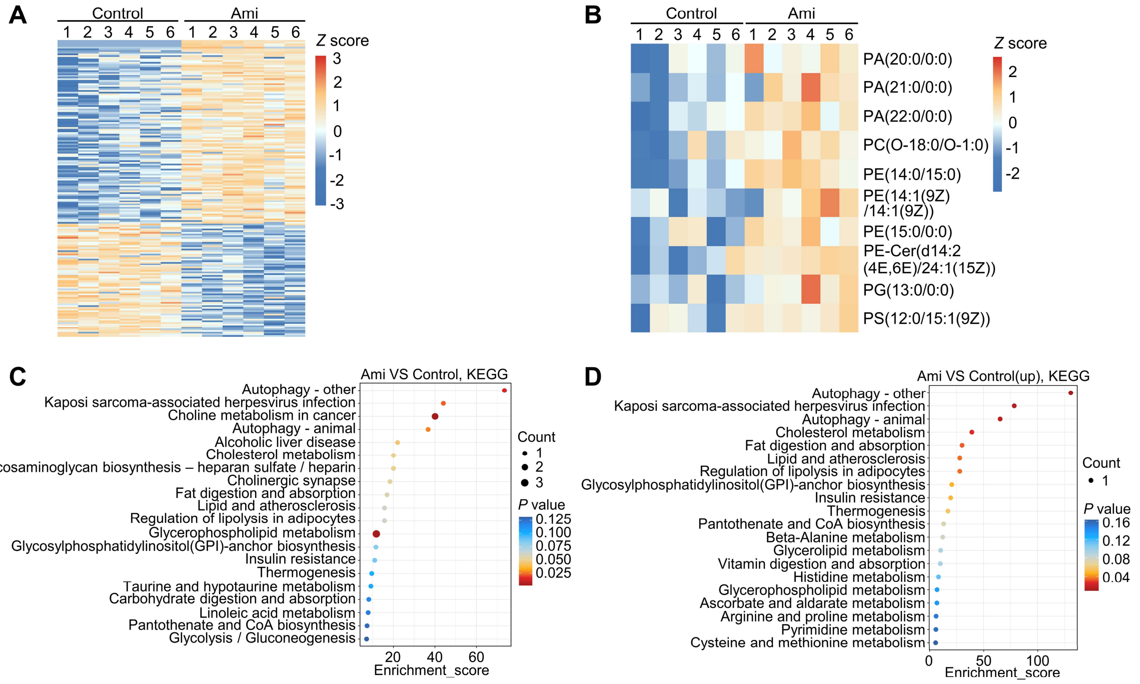

Figure S10. Heatmap and KEGG analysis of differential metabolites in granulosa cells from naturally aged mice. The naturally aged mice were administered water either with or without 4.0 mM aminophylline, and then the metabolome of granulosa cells was examined. **A-B**, Heatmap showed differential metabolites in granulosa cells from control and aminophylline (Ami) groups. **C-D**, The top twenty altered (**C**) and upregulated (**D**) pathways in granulosa cells from aminophylline group compared to those from control group by KEGG analysis.

Table S1. List of primary antibodies used in this study. IF: Immunofluorescence; WB: Western blotting.

Antibody	Catalog Code	Source	Host	Dilution	
				IF	WB
Akt	4691	Cell Signaling Technology	Rabbit	__	1:1000
p-Akt	4060	Cell Signaling Technology	Rabbit	1:200	1:1000
BrdU	ab1893	Abcam	Sheep	1:200	__
BAX	50599-2-Ig	Proteintech	Rabbit	__	1:1000
BCL2	26593-1-AP	Proteintech	Rabbit	__	1:1000
Cleaved Caspase-3	9664	Cell Signaling Technology	Rabbit	1:50	1:1000
DDX4	ab27591	Abcam	Mouse	1:200	1:1000
FOXL2	NB100-1277	Novus Biologicals	Goat	1:300	__
FOXO3a	12829	Cell Signaling Technology	Rabbit	1:100	1:1000
GDF9	ab254323	Abcam	Rabbit		1:1000
GLUT4	ab33780	Abcam	Rabbit	__	1:1000
HK1	ab150423	Abcam	Rabbit	__	1:1000
Ki-67	9129s	Cell Signaling Technology	Rabbit	1:100	__
mTOR	2972	Cell Signaling Technology	Rabbit	__	1:1000
p-mTOR	2971	Cell Signaling Technology	Rabbit	1:200	1:1000

PCNA	2586	Cell Signaling Technology	Mouse	1:100	1:1000
PFKL	ab181064	Abcam	Rabbit	__	1:1000
PKM2	4053	Cell Signaling Technology	Rabbit	__	1:1000
ZP3	sc-398359	Santa Cruz	Mouse		1:1000
α-Tubulin	ab195887	Abcam	Mouse	1:300	__
β-actin	4967	Cell Signaling Technology	Rabbit	__	1:1000

Table S2. Primers for qRT–PCR used in this study.

Genes	Forwards (5'-3')	Backwards (5'-3')
Aldoa	CGTGTGAATCCCTGCATTGG	CAGCCCCTGGGTAGTTGTC
Bax	TTTCATCCAGGATCGAGCAGG	GCAAAGTAGAAGAGGGCAACCAC
Bcl2	CTACCGTCGTGACTTCGCA	TACCCAGCCTCCGTTATCC
Caspase-3	CCGGTTACTATTCCTGGAGA	TAACACGAGTGAGGATGTGC
Eno1	TGCGTCCACTGGCATCTAC	CAGAGCAGGCGCAATAGTTTTA
Gdf9	TCTTAGTAGCCTTAGCTCTCAGG	TGTCAGTCCCATCTACAGGCA
Glut4	ACACTGGTCCTAGCTGTATTCT	CCAGCCACGTTGCATTGTA
Hk1	CGGAATGGGGAGCCTTTGG	GCCTTCCTTATCCGTTTCAATGG
Ki-67	ATCATTGACCGCTCCTTTAGGT	GCTCGCCTTGATGGTTCCT
Ldhb	CATTGCGTCCGTTGCAGATG	GGAGGAACAAGCTCCCGTG
Pcna	CGGCGTGAACCTGCAGAGCA	GGTTGCGGTCGCAGCGGTAT
Pde1a	CCAGACTGACTCCGTCCCAT	CCATTTTGCGTGTGAAAGTTGA
Pde1b	GATGCTGGAGTCGGATTGCC	TTCAGTGTCTAGGATTTGCCTTG

Pde1c	ACGTCCCAGAGGTTACGGT	GGCTGCATATTCCAGATTCTTCT
Pde2a	TGGCGTTGTGGACGATGAG	CGCGATAGAAAAGCGGATGG
Pde3a	CCTGGACTAGCGTGCTTAGGA	CAGGCGACCTTGAACCTCT
Pde3b	AAAGCGCAGCCGGTTACTAT	CACCACTGCTTCAAGTCCCAG
Pde4a	AATGCCCTACAGACGCCTG	GACGGTGTTGGCCCATTTT
Pde4b	TTCACGGTGGCTCATACATGC	CGCTGTCAAGATCGTAGAGGAA
Pde4c	TCCGAGAGCCAGTGGATTCT	CCTTGAGTTCCAATCGTGAAGAC
Pde4d	TTTTGCCAGTGCAATACATGATG	CAGAGCGAGTTCCGAGTTTGT
Pde5a	CGGCCTACCTGGCATTCTG	GCAAGGTCAAGTAACACCTGATT
Pde6a	CAGCAACTACCACGATGTGAA	GTGGACATTGAAGAGCCTAGTG
Pde6b	GCAGCACTTTTTGAACTGGTG	CATTGCGCTGGCGGTACATA
Pde6c	GCGGCAGTTTGAAACGGTG	CATCATAGGCTGACTCTGCAC
Pde6d	CCCGTGTGCCCAAGAAAATC	CCACTCTTCTAGGCATTGTCCTT
Pde6g	AGGGTGAGATTCGGTCAGC	GCTCTTGAACTGCCTTGTTTG
Pde6h	AGGGGTGAAAGGGTTTGGAGA	ATGATCCCGAACTGAGCAAGC
Pde7a	AGTGGATCACCTCTAAGAGACG	CGGACATCTCCTAGCATACGAAT
Pde7b	TGCTAGGAGATGTACGACTAAGG	GGGCCTGCGGTATAATCCC
Pde8a	CCGAGCATCCACACTTCCG	TCAGCTACTGATACCTTCGAGG
Pde8b	AGAGCGGTGTGATCTACTGC	CGTCGGTCTGCACGAAGAG
Pde9a	CCACCATCTCCCTTTTAACCAC	CAGCACGCCCTGGATAAGT
Pde10a	GCAGGGGACAATCCTTGCC	CGTCAAACCTCTTGTGAACTCTT
Pde11a	AACAGGACCTACGATGAACAGG	TGAGGCAGATTCACCCTCGAT
Pfkl	GAACTACGCACACTTGACCAT	CTCCAAAACAAAGGTCCTCTGG

Rpl19	CTGAAGGTCAAAGGGAATGTGTTC	TGGTCAGCCAGGAGCTTCTTG
Tpi	CCAGGAAGTTCTTCGTTGGGG	CAAAGTCGATGTAAGCGGTGG
Zp3	CCTCAGGACTAACCGTGTGGA	CCATCAGGCGAAGAGAGAAAG
